# *Streptococcus pneumoniae* adaptation to nutrient deprivation and immune modulation drives upper respiratory tract colonization

**DOI:** 10.1093/ismejo/wrag213

**Published:** 2026-08-19

**Authors:** Daan W Arends, Kristin Surmann, Lucille F van Beek, Robert S Jansen, Rob J Mesman, Jeroen D Langereis, Monique van Scherpenzeel, Manuela Gesell Salazar, Uwe Völker, Gerco den Hartog, Marien I de Jonge

**Affiliations:** Centre for Immunology of Infectious Diseases and Vaccines, National Institute of Public Health and the Environment, 3720 BA Bilthoven, The Netherlands; Laboratory of Medical Immunology, Radboud University Medical Centre, 6500 HB Nijmegen, The Netherlands; Department of Functional Genomics, Interfaculty Institute for Genetics and Functional Genomics, University Medicine Greifswald, 17475 Greifswald, Mecklenburg-Vorpommern, Germany; Laboratory of Medical Immunology, Radboud University Medical Centre, 6500 HB Nijmegen, The Netherlands; Department of Microbiology, Radboud Institute for Biological and Environmental Sciences, Faculty of Science, Radboud University, 6525 AJ Nijmegen, The Netherlands; Department of Microbiology, Radboud Institute for Biological and Environmental Sciences, Faculty of Science, Radboud University, 6525 AJ Nijmegen, The Netherlands; Laboratory of Medical Immunology, Radboud University Medical Centre, 6500 HB Nijmegen, The Netherlands; GlycoMScan, 6827 DG Arnhem, The Netherlands; Department of Functional Genomics, Interfaculty Institute for Genetics and Functional Genomics, University Medicine Greifswald, 17475 Greifswald, Mecklenburg-Vorpommern, Germany; Department of Functional Genomics, Interfaculty Institute for Genetics and Functional Genomics, University Medicine Greifswald, 17475 Greifswald, Mecklenburg-Vorpommern, Germany; Centre for Immunology of Infectious Diseases and Vaccines, National Institute of Public Health and the Environment, 3720 BA Bilthoven, The Netherlands; Laboratory of Medical Immunology, Radboud University Medical Centre, 6500 HB Nijmegen, The Netherlands; Laboratory of Medical Immunology, Radboud University Medical Centre, 6500 HB Nijmegen, The Netherlands

**Keywords:** *S. pneumoniae*, upper respiratory tract adaptation, immune evasion, colonization, nasal epithelium, proteomics, metabolomics, cytokines, *in vivo*-mimicking medium

## Abstract

*Streptococcus pneumoniae* is a successful colonizer of the human upper respiratory tract; however, the mechanisms that enable its persistence in this nutrient-limited environment, with numerous immune mechanisms in place, remain enigmatic. Here, we examined how pneumococci adapt to upper respiratory tract conditions and how this affects host interactions. We measured intranasal metal ion and monosaccharide concentrations to create an *in vivo-*mimicking medium for studying pneumococcal adaptation. Growth in this medium was reduced compared to glucose-rich chemically defined media (CDM). Proteome analysis revealed a shift to galactose as the major carbohydrate source, and decreased levels of fatty acid biosynthesis proteins and pneumolysin, compared to other CDMs. Glycerophosphocholine accumulated extracellularly leading to decreased C-reactive protein and Immunoglobulin M binding to pneumococci. Pneumococci grown in *in vivo*-mimicking medium, compared to glucose-rich media, were more capable colonizers of primary epithelium and induced less epithelial cytokine release. Together, this shows how pneumococci adapt to the nutrient-limited respiratory environment, modulate epithelial cells, and evade humoral responses to facilitate persistent colonization.

## Introduction

The upper respiratory tract (URT) of humans is the only natural environment in which *Streptococcus pneumoniae*, the pneumococcus, thrives. Despite being an inhospitable environment low in freely available nutrients and with a plethora of antimicrobial mechanisms in place, the pneumococcus reaches carriage rates of 25%–80% in 0–5-year olds [1–5], ~30% in parents and <10% in childless adults [6]. Colonization persists for a duration ranging from ~5 to 30 weeks [7]. Although colonization of the URT is mostly asymptomatic, pneumococci can cause severe disease by invading the bloodstream or colonizing the lower respiratory tract; it is the most common cause of bacterial pneumonia [8]. This results in over 1 million annual deaths, including an estimated 300 000 deaths in <5-year olds [9, 10], and many more non-lethal severe infections.

Pneumococcal persistence in the human host requires adaptation to the URT environment. The mucus covering the epithelium is low in free nutrients, due to active sequestration, transportation, and degradation [11, 12]. To acquire essential metal ions, the pneumococcus expresses multiple ABC transporters [13]. In the URT, concentrations of readily metabolizable carbohydrates are low; most available carbohydrates are present in complex forms attached to mucins, the major components of the mucus covering the epithelial surface [14]. Mucins are large, secreted or membrane-attached proteins that are highly glycosylated [14]. MUC5B and MUC5AC are the most prevalent secreted mucins in the URT [14]. Their glycans consist of multiple types of monosaccharides that are capped with sialic acids (Neu5Ac) or sulfate groups [15]. To access these monosaccharides, the pneumococcal genome encodes multiple neuraminidases to remove the terminal Neu5Ac [16]. Additionally, it contains multiple (predicted) enzymes that can break glycosidic bonds within the glycans, and transporters importing the resulting monomers/polymers into the bacterial cell [16]. Besides nutritional challenges, mucociliary clearance and the presence of antimicrobial immune factors present in mucus necessitate effective adhesion to epithelial surfaces and require mechanisms to withstand host immune factors [10].

The upper airway epithelium plays a central role in the recognition and subsequent response to potential pathogens. By recruitment and activation of immune cells and the induction of inflammatory responses, or refraining from these responses, a tolerant or anti-microbial reaction is generated upon pathogen recognition. *In vitro* studies on epithelial cytokine responses have shown the induction of CXCL2, CXCL8, CCL2, CCL20, IL-6, and IL-1β upon pneumococcal colonization [17, 18]. Pneumococcal factors have been shown to affect epithelial cytokine release, such as the pore-forming toxin pneumolysin (Ply), which induces CXCL8 release by Detroit 562 cells [19, 20]. However, despite its evident threat to human health, pneumococcal colonization of the URT induced relatively little epithelial immune responses, compared to *Neisseria meningitidis* or respiratory syncytial virus [21], likely facilitating its persistence. Whether this limited immune response reflects active repression of the immune system by the pneumococcus is unclear.

In this study, we investigated how pneumococci achieve this persistent colonization by examining their adaptation to URT conditions. For this purpose, pneumococci were cultured in media closely mimicking *in vivo* nutrient availability, to assess their growth, polysaccharide capsule morphology, extracellular metabolites, and proteome. Finally, we evaluated whether adaptation to URT conditions affected subsequent epithelial infection and evasion of humoral immunity.

## Materials and methods

### Ethics statement

Nasal fluid collection from in total 10 healthy adults for measurements of sugar levels was approved by the medical ethical committee of the Radboud University Medical Centre Nijmegen and written informed consent was obtained from all volunteers. Volunteers were excluded based on the following criteria: symptoms of a recent common cold, recent nose bleedings, or frequent contact with young children. Nasal respiratory epithelial cells were collected as described previously [21]. In brief, macroscopically unaffected surgery samples were obtained under protocols approved by the relevant ethics committee.

### Nasal nutrient measurements and pneumococcal culture conditions

To determine monosaccharide concentrations, nasal fluid from ten healthy adults was collected in summer by incubating Schirmer strips (Daxtrio, Zaandam, The Netherlands) for 1 min in the inferior turbinate. Further details are described in the supplementary methods. Metal ion measurements were performed previously as described in van Beek *et al.* 2020 [22]. The same metal ion concentrations were used in the present study.

Growth media used were Todd Hewitt broth with 0.5% yeast extract (THY), chemically defined medium (CDM) as described by Kloosterman *et al.* 2006 [23], CDM with nasal metal ion concentrations as measured by Van Beek *et al.* [22] (CDM + M), and CDM + M additionally supplemented with the monosaccharides at geometric mean concentrations we measured *in vivo* and supplemented with mucin (MUC2) from the porcine stomach (Sigma) (CDM + MS). Dissolved mucin was dialyzed using a Snakeskin dialysis membrane with a 10 kDa cutoff (Thermo Fischer Scientific) to remove free sugars. See Supplementary Table 4 for detailed CDM medium constitutions.

In our experiments, carriage isolates BHN418 (serotype 6B) and BHN100 (19F) were used. For epithelial infection experiments, mutants of BHN418 and BHN100 with a deleted capsule polysaccharide gene locus (Δ*cps*) were also used. To assess the growth in the different media, 10 ml of pre-warmed medium was inoculated with 1–5^*^10^6^ CFU/ml of WT BHN100 or BHN418 grown in corresponding medium (see supplementary methods for preculturing details). At 2-h increments, growth was assessed using optical density measurements at 620 nm wavelength (OD), colony forming unit (CFU) count of serial dilutions of 15 μL of bacterial cultures on Columbia 5% sheep blood agar plates, and flow cytometry event count. For flow cytometry, 200 μL of bacterial culture was centrifuged at ~15 000 × g for 10 min. After removal of supernatant, samples were fixed in 2% PFA (PFA) at room temperature (RT; ~20°C) for 20 min. Pneumococci were stained with 10 μM Pacific Blue Succinimidyl Ester (PBSE). PBSE-positive events were counted using a CytoFLEX (Beckman Coulter Life Sciences). All growth curve replicates (*n* = 4–7) were sampled on different days. In separate experiments, transmission electron microscopy (TEM) samples were collected at 3 h or 8 h after inoculation. Further details of sample treatment and image acquisition are described in supplementary methods.

### Proteomics sample harvest and data acquisition

Tubes of 10 ml of preheated CDM, CDM + M, and CDM + MS were inoculated with 1–5^*^10^6^ CFU/ml BHN100 or BHN418 grown in the corresponding medium. Samples were collected after 2 h (exponential phase in all conditions), 6 h (stationary phase for BHN100 grown in CDM or CDM + MS) or 8 h (stationary phase for BHN100 grown in CDM + M and all BHN418 cultures) of growth. Based on the growth curves, the volume that should contain 1^*^10^8^ CFU was centrifuged for 10 min at ~1900 × g. Bacterial pellets were resuspended in 100 μL HEPES (20 mM; pH 8.0) with 5% (m/v) sodium dodecyl sulfate without ethylenediaminetetraacetic acid and incubated at 95°C for 1 min and stored at −80°C. At time of collection, OD620, CFU, and flow cytometry event count were determined as well. Four biological replicate samples were taken, each on a separate day, for each described combination of strain, medium and growth phase. Prior to measuring, bacterial cells were disrupted and proteins were digested using tryptic digestion (supplementary methods).

During nano-liquid chromatography–tandem mass spectrometry (LC–MS/MS) analysis, tryptic peptides were first separated on an Ultimate 3000 nano-LC system (Thermo Fisher Scientific, USA) and subsequently analyzed on an Orbitrap Exploris 480 mass spectrometer (Thermo Fisher Scientific, USA) in data-independent acquisition (DIA) mode. MS settings are summarized in Supplementary Table 5. Further data processing is described in supplementary methods. The MS proteomics data and the established database have been deposited to the ProteomeXchange Consortium via the PRIDE [24] partner repository with the dataset identifier PXD072730.

### Exometabolome analysis

In three independent experiments, 10 ml of preheated CDM or CDM + MS was inoculated with 1^*^10^6^ CFU/ml BHN100 or BHN418 and incubated at 37°C 5% CO_2_. At t = 0 h and t = 4 h, supernatant was collected and sterilized using a 0.2 μm filter. As a control, uninfected CDM/CDM + MS was incubated for 4 h at 37°C 5% CO_2_ and sterilized. A total of 100 μL of sterilized sample was mixed with 400 μL acetonitrile:methanol (1:1) and incubated at 4°C for 10 min. Then, samples were centrifuged at ~15 000 rpm for 10 min 4°C and stored at −80°C. Blanks consisting of the acetonitrile:methanol mixture were also included in the analysis. For each condition, three biological replicate samples were measured. LC–MS measurements and further analyses are described in supplementary methods. Dataset has been deposited in the MassIVE repository with the dataset identifier MSV000100798.

### C-reactive protein and immunoglobulin M binding to pneumococci in the presence of cholines

We adapted a previously described protocol [25] replacing *Haemophilus influenzae* with our BHN418 WT and Δ*cps* strains. Heat-inactivated normal human serum (HI-NHS) was prepared by incubating NHS at 56°C for 20 min to inactivate complement. Hank’s balanced salt solution (HBSS) with Ca^2+^ and Mg^2+^ was supplemented with 0.1% bovine gelatin (HBSS3+). Dilution series of choline (Cho) chloride, phosphocholine (Pcho) chloride calcium salt tetrahydrate or L-α-glycerophosphocholine (GPC) in HBSS3+ with 10% HI-NHS were made, from 10 mM to 40 μM in 1:5 serial dilutions. THY-grown BHN418 WT and Δ*cps* glycerol stocks were washed and diluted in HBSS3+ to an OD620 of 0.1. A total of 50 μL of bacterial suspension was mixed with 50 μL of serum-Cho solution and incubated for 30 min at 37°C. Then, samples were centrifuged at 3.200 × g and fixed in 2% PFA for 20 min at RT. Afterwards, C-reactive protein (CRP) and immunoglobulin M (IgM) were stained using 1:500 polyclonal goat α-human CRP (Acris Antibodies) followed with 1:1000 FITC-polyclonal donkey α-goat IgG (ThermoFisher), and 1:1000 Alexa Fluor 647-rabbit α-human IgM (Jackson Immunoresearch) respectively. Antibodies were diluted in phosphate-buffered saline (PBS) + 2% bovine serum albumin for 30 min at RT. Bacteria were stained with by incubation with SYTO40 nucleic acid stain (ThermoFisher Scientific; 5 μM) for 5 min. Samples were washed in between steps with 2 × 100 μL PBS. Samples were measured using a CytoFLEX (Beckman Coulter Life Sciences). Data were attained in three independent experiments where each condition was measured in duplo or singly.

### Pneumococcal infection of differentiated epithelium

Approximately 1^*^10^6^ CFU/ml BHN418 WT or the unencapsulated mutant BHN418 Δ*cps* were cultured in CDM, CDM + M, CDM + MS, or THY for 4 h and stored in 15% glycerol −80°C. Frozen stocks were thawed and CFU of stocks was determined. Medium of 4–6-week-old differentiated epithelial cell layers (see supplementary materials for details on epithelial culturing) was replaced with differentiation medium without penicillin/streptomycin and heparin. After three days, medium was replaced again without antibiotics and heparin, and the apical side was washed with 100 μL PBS and transepithelial resistance was determined (TEER). The amount of pneumococcal stock required to infect a number of inserts with ~1^*^10^6^ CFU was centrifuged and resuspended in 25 μL PBS per insert to be infected. To the apical side of each insert 25 μL of inoculum was added: CDM/CDM + M/CDM + MS/THY-grown pneumococci in 25 μL PBS, or 25 μL mock inoculum consisting of a 1:10 dilution of sterilized CDM/CDM + M/CDM + MS/THY in PBS. For each infection condition, 12 inserts were used per epithelial cell donor, totaling 36 inserts per infection condition. In total, 8 inserts were used per donor for uninfected inserts, totaling 24 inserts for each uninfected condition. Inoculated epithelial cell layers were incubated at 37°C for 48 h. After 48 h, epithelial cell layers were washed apically with 100 μL PBS, TEER was measured, and the epithelial layer was sacrificed for CFU measurements by mechanically scraping the cells from the membrane and homogenizing the resulting suspension through repeated pipetting.

CFU of the infection inoculum and the individual 48 h samples were measured by counting CFUs of serial dilutions cultured on Columbia 5% sheep blood agar plates. TEER was measured using a Millicell ERS-2 Volt-Ohm Meter (Merck), by adding 100 μL PBS apically and measuring the resistance (Ω) across the cell layer. Resistance of empty inserts was subtracted from measurements. For Fig. 5, TEER fold change between 0 h and 48 h was determined. For cytokine measurements, 50 μL basolateral samples were taken at 0 h, 24 h and 48 h. Apical cytokines were measured in the 100 μL PBS added at 48 h for TEER measurement. Further details are described in supplementary methods.

## Results

### Upper respiratory tract nutrient composition affects pneumococcal growth

**Figure 1:**
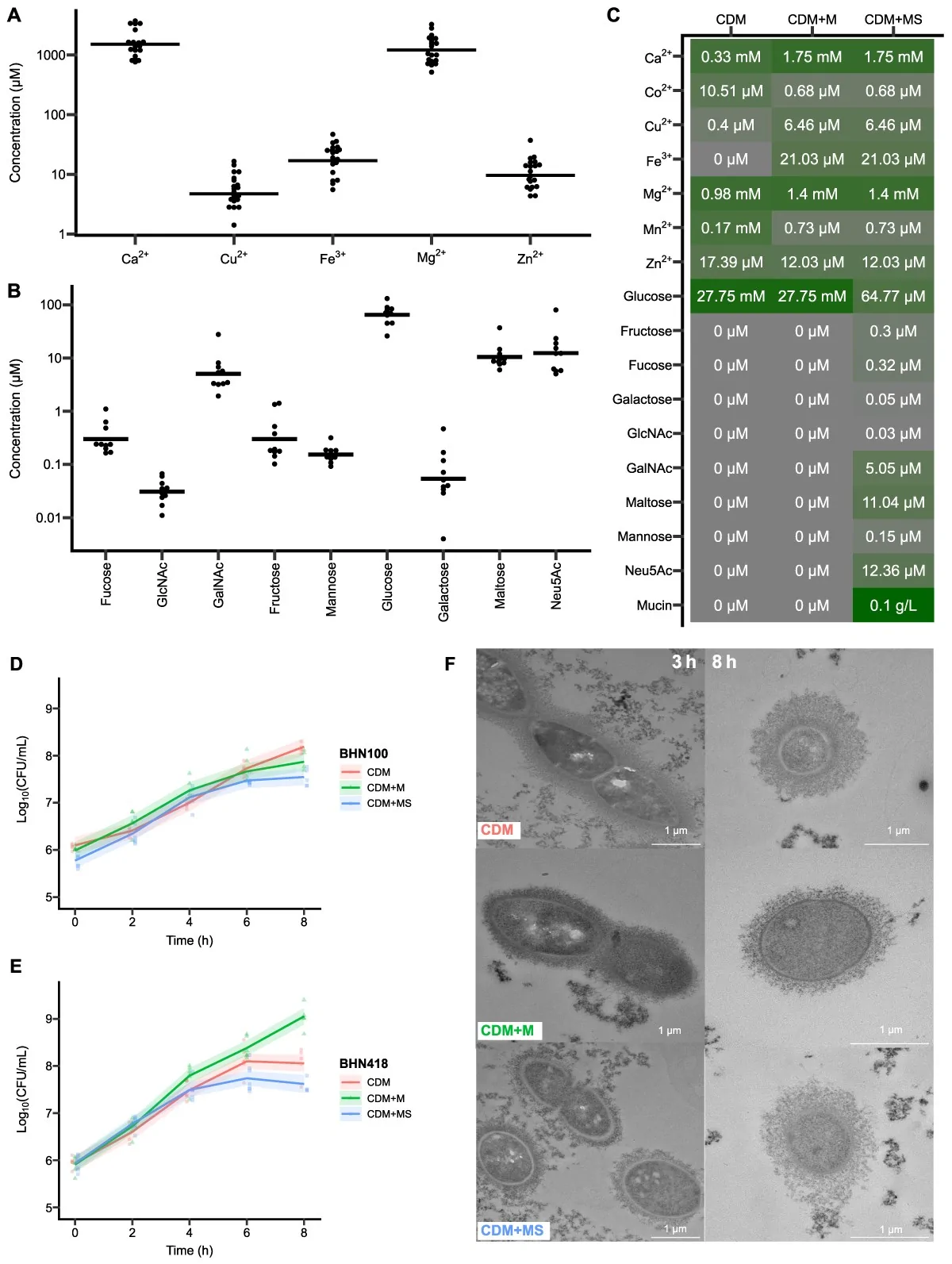
Mimicking the URT nutrient composition reduces pneumococcal growth with minor effects on capsule macrostructure. Mass spectrometry measurements of metal ion (A) and monosaccharide (B) concentrations in nasal lining fluids of healthy adults (panel a data are derived from van Beek *et al.* 2020 [22]) were used to adapt CDM [23]. This resulted in a CDM with *in vivo* metal ion concentrations (CDM + M), and a CDM with metal and sugars in *in vivo* concentrations (CDM + MS). Mn^2+^ and Co^2+^ were not detected, and their concentrations were set at the detection limits (see van Beek *et al.* 2020 for more details). Plots show the measurements of nasal fluids of 10 individuals measured in summer and winter (a; *n* = 20), or summer only (b; *n* = 10). Crossbars indicate geometric mean concentrations. (C) Comparison of metal ion and monosaccharide concentrations in the three CDMs used in experiments. Green coloration indicates higher nutrient concentration. Growth curves of BHN100 (D) and BHN418 (E) in CDM, CDM + M, or CDM + MS, based on CFU (CFU/ml) counts at indicated timepoints. Lines represent the estimated marginal mean of a linear mixed-effects model with 95% confidence intervals. Dots represent biological replicates (*n* = 4–7). (F) Electron microscopy images of pneumococci (BHN418) after 3 h (left) or 8 h of growth (right) in the different media.

To investigate the impact of the URT microenvironment on pneumococci we further improved a physiological CDM [23], which we had previously adapted to contain metal ion concentrations as measured in nasal fluid (CDM + M; see reference 22) [22] (Fig. 1A). Both CDM and CDM + M contain glucose as only carbohydrate source, which does not reflect the URT environment. Because monosaccharide concentrations in nasal fluid were not determined previously, we measured these to further adapt the medium to the *in vivo* composition (Fig. 1B). Medium containing solely *in vivo* monosaccharide concentrations as a carbohydrate source did not support pneumococcal growth (Supplementary Fig. 1a). However, supplementation of mucin (0.1 g/L) to CDM with *in vivo* concentrations of metal ions and sugars (CDM_MS), enabled pneumococcal growth. Porcine stomach mucin was used, which consists mainly of Muc5ac which was shown to contain similar glycans as MUC5B in human saliva [26]. Differences in metal ion and monosaccharide concentrations in CDM, CDM + M, and CDM + MS are shown in Fig. 1C.

First, we assessed the growth of two pneumococcal isolates, BHN100 and BHN418, when cultured in CDM, CDM + M, or CDM + MS by measuring CFUs for 8 h (Fig. 1D and E). CDM + MS showed significantly lower mean CFUs and area under the curve after 8 h (Supplementary Fig. 1b), at which time all comparisons differed significantly (*P* < .05) for both strains. Overall, medium, time, strain, and their two- and three-way interactions significantly affected Log_10_CFU (*P* < .001, type III analysis of variance). Although both strains showed similar growth in CDM and CDM + MS, CDM + M greatly increased growth of BHN418, but not BHN100, suggesting the effect of changes in metal ion concentration is more strain dependent. Flow cytometry events, an alternative method to determine bacterial counts, and OD_620_ measurements of growth curves followed the same pattern as CFU measurements (Supplementary Fig. 1c and d).

The pneumococcal polysaccharide capsule is energy-expensive to synthesize, which was shown to affect pneumococcal growth in nutrient limiting conditions [27]. TEM was used to assess the impact of physiological conditions on the pneumococcal polysaccharide capsule at 3 h and 8 h (Fig. 1F). At 3 h, we observed the highest capsule thickness in CDM, followed by CDM + M and lowest in CDM + MS. BHN418 grown in CDM + MS had the most irregular capsule structure and CDM the most regular. At 8 h, capsule thickness was more heterogeneous in all samples, however, growth in CDM and CDM + MS generally resulted in a thicker capsule compared to CDM + M (Fig. 1F). Taken together, these results show that nutrients as measured *in vivo* affect pneumococcal growth, and suggest that at higher bacterial loads certain nutrients become limiting. Capsule thickness was dependent on initial nutritional environment, as well as growth phase and associated depletion of nutrients. It is unclear whether the observed differences are a result of direct effects of certain nutrients on capsule regulation, or differences in growth speed. Pneumococci can form chains upon repeated replication without complete separation, and increased chain length has been associated with improved adherence [28]. In the TEM images, most pneumococci were observed individually or as a diplococcus, with little differences between the media.

### Upper respiratory tract conditions induce changes in pneumococcal carbohydrate metabolism, fatty acid biosynthesis, and pneumolysin levels

**Figure 2:**
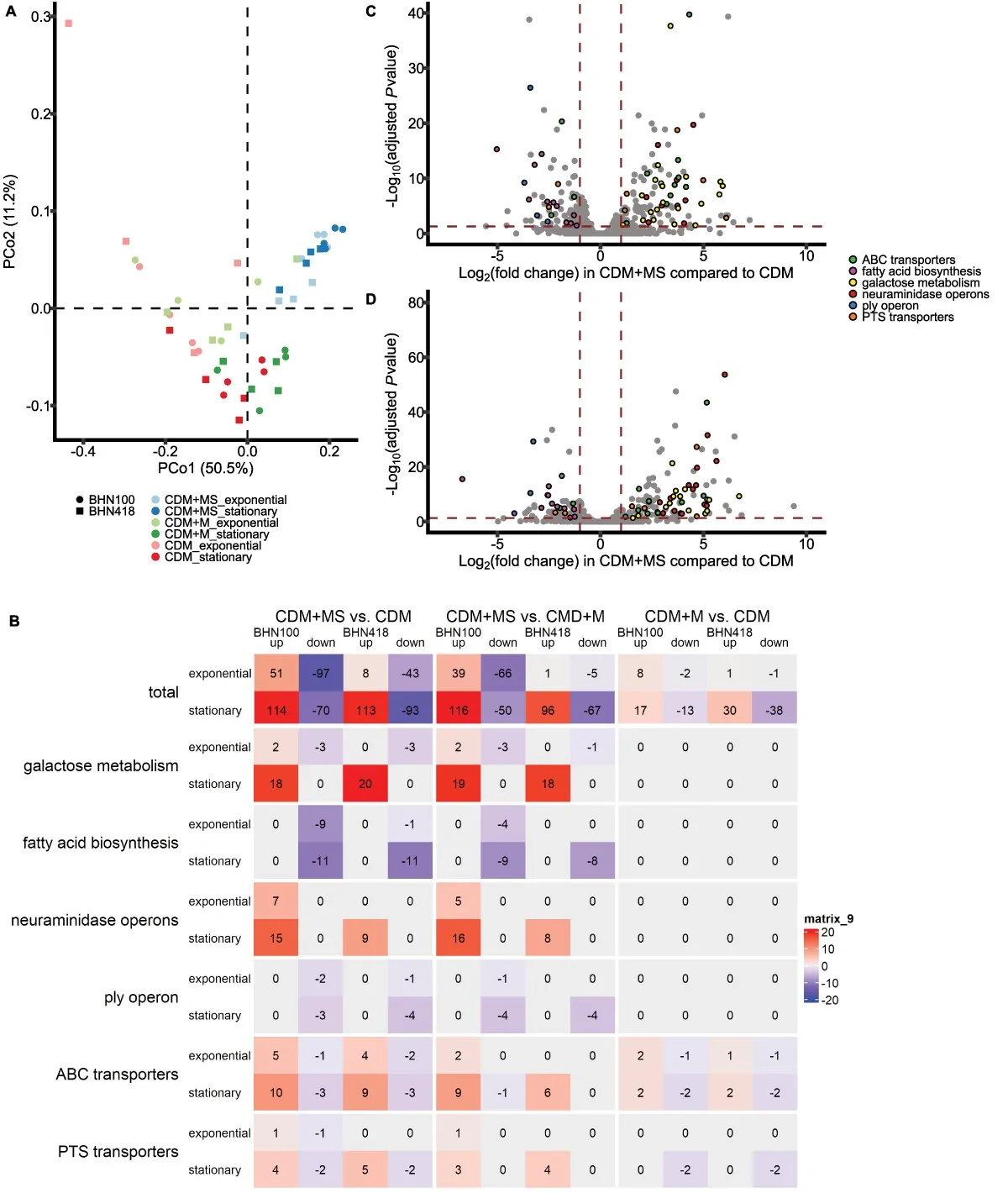
URT conditions alter pneumococcal metabolism and reduce Ply levels. Proteome analysis of BHN100 and BHN418 grown in CDM, CDM + M, and CDM + MS in quadruplicate independent experiments collected during exponential growth (t = 2 h) and stationary phase (t = 6–8 h). (A) Bray–Curtis dissimilarity based principal coordinate analysis of proteomes in different growth media (*n* = 4). Dot color and shape indicate growth medium, pneumococcal strain, and growth phase. (B) Heatmap displaying differentially regulated protein groups in the different media. Number of proteins present in lower and higher abundance for each grouping is shown for each strain and growth phase. Individual proteins associated with each group can be found in Supplementary Table 1. (C and D) Volcano plots showing protein fold-change differences and *P*-value between CDM and CDM + MS in stationary phase of BHN418 (C) and BHN100 (D). Individual protein fold changes and *P*-values, and associated higher or lower abundance were determined by performing ROPECA statistics (*n* = 4).

To further analyses the impact of the nutrient availability of the URT on pneumococci, we performed proteome profiling of BHN418 and BHN100 cultured in CDM, CDM + M or CDM + MS during exponential growth (at 2 h) and stationary phase (6–8 h). Proteome profiling was carried out using a DIA mass spectrometry–based proteomics approach. Using a pangenome combining BHN100 and BHN418 protein amino acid sequences, protein abundances were quantified using maxLFQ [29], which estimates protein intensity based on normalized peptide signals across samples. In total 1447 unique proteins could be detected across all samples, with protein counts ranging between 750 and 1250 for most samples. Rarefaction curves of accumulated DIA quantified precursor features, evaluating proteomics sampling depth across different conditions, indicated comparable sampling saturation for the stationary phase samples (Supplementary Fig. 2a). Exponential phase conditions showed more varying saturation, potentially reflecting differences in proteome complexity, abundance distributions, and/or effective sampling depth between conditions. Most detected proteins were found in all conditions, but some condition-specific differences could be observed (Supplementary Fig. 2b). Principal coordinate analysis of maxLFQ proteins intensities revealed that CDM + MS samples from both growth phases and isolates grouped separately from CDM and CMD + M (Fig. 2A), suggesting an altered proteome in CDM + MS, even when compared to the stationary phase samples of the other media, where nutrient-depletion could also induce alternative carbohydrate-metabolic pathways. Principal component analysis showed a similar pattern, with a clearer separation between strains along PC2 (Supplementary Fig. 3a).

Next, we compared individual protein level differences between conditions via reproducibility optimized peptide change averaging (ROPECA) statistics [30]. The largest differences were observed during the stationary phase of CDM + MS-grown and CDM-grown pneumococci (Fig. 2B), with 184 (BHN100) or 206 (BHN418) proteins displaying different levels between both conditions (Log_2_ fold change >1 or <−1, adjusted *P* < .05), of which 107 were influenced in both strains. Gene ontology enrichment analysis of differentially abundant proteins in this comparison revealed a clear enrichment of carbohydrate metabolism and transport processes (Supplementary Fig. 3b and c, Supplementary Table 2 and 3). Based on this analysis, additional STRINGdb [31] clustering and identification of (predicted) operons using PneumoBrowse2 [32], we identified several differentially regulated groups (Fig. 2B–D, Supplementary Table 1). In CDM + MS, a clear utilization of galactose was observed, with an increase of protein levels from both the Leloir- and tagatose–galactose metabolism pathways. Additionally, an increase of abundance was observed for proteins of neuraminidase-operons involved in the removal of Neu5Ac from mucins and subsequent Neu5Ac metabolism. Levels of multiple ABC-transport system proteins and phosphotransferase system transporter proteins involved in carbohydrate were increased as well, suggesting a broadening of nutrient acquisition from the environment. Besides changes in carbohydrate-metabolism and uptake, a clear downregulation of fatty acid biosynthesis (FASII) was observed, with lower levels of proteins involved in the synthesis of acyl-acyl carrier protein from acetyl-CoA [33]. The observed decrease in levels of FabM and FakB3, responsible for the production or uptake of unsaturated fatty acid [34, 35], is expected to result in an increased saturated:unsaturated fatty acid ratio, affecting membrane fluidity.

Apart from metabolic changes in CDM + MS, we also observed a downregulation of Ply and hypothetical proteins in the same operon in URT conditions.

### Glycerophosphocholine accumulates extracellularly in upper respiratory tract conditions, inhibiting C-reactive protein/immunoglobulin M binding

**Figure 3:**
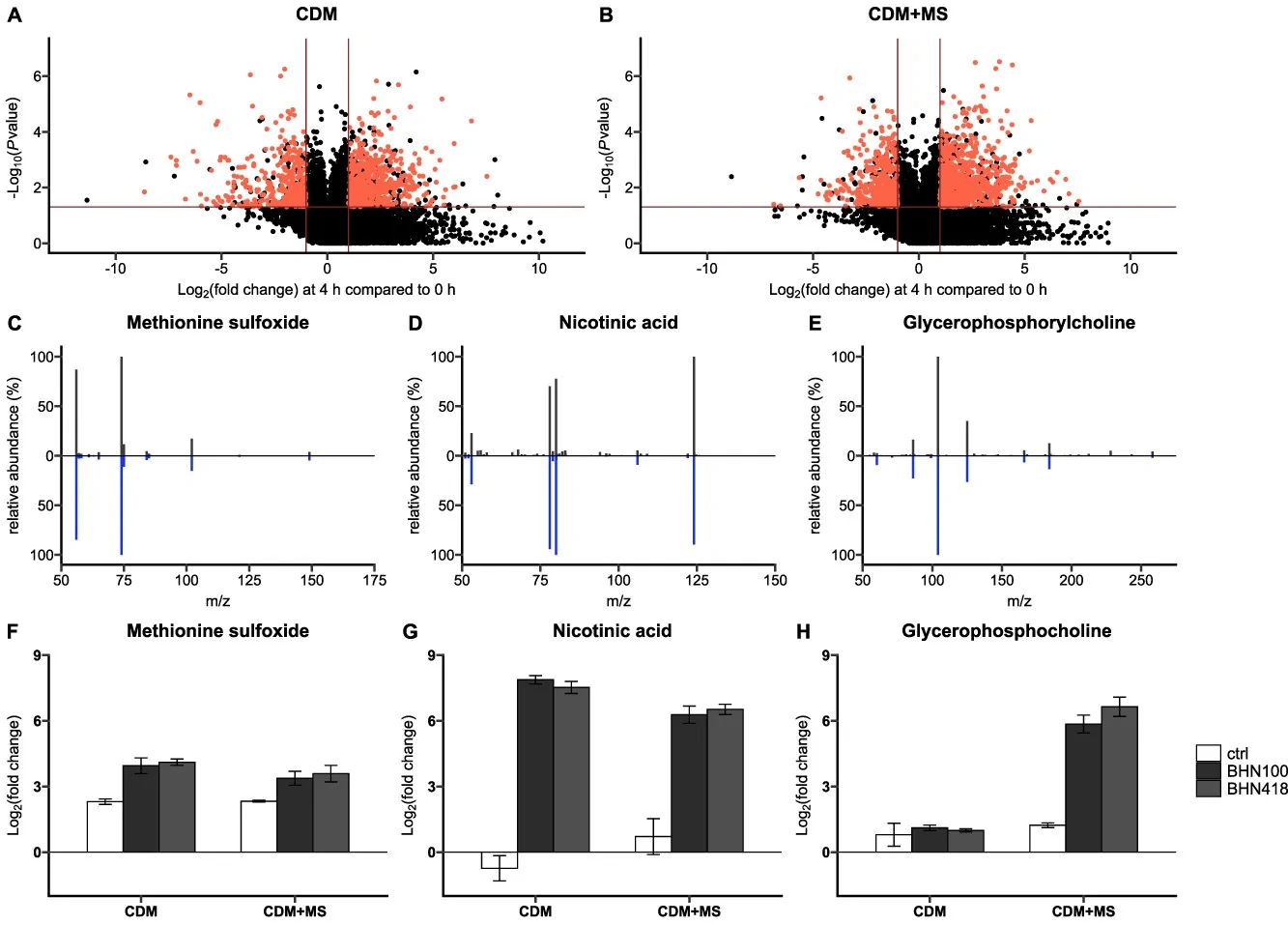
GPC accumulates in pneumococcal CDM + MS cultures. Exometabolomic analysis of BHN100 and BHN418 grown in CDM or CDM + MS. Culture supernatants were collected at 0 and 4 h in three independent experiments and filtered through 0.2 μm filters. (A and B) Volcano plots of detected features in BHN418 CDM (A) and CDM + MS (B) supernatants using untargeted mass spectrometry in the positive ionization mode. Log_2_ fold change between 0 and 4 h is shown. Lines represent cut off values for features displaying significantly increased or decreased intensity. Red indicates features that also displayed increased/decreased intensities at 4 h vs. 0 h in BHN100 culture supernatants. (C–E) MS^2^-spectra of three metabolites that increased over 4 h in the supernatant (black) compared with those of reference standards of the respective metabolite (blue). (f–h) Specific Log_2_ intensity fold changes of the three identified metabolites in BHN100 and BHN418 cultures. Control bars show changes in metabolite concentration in medium without bacteria incubated under the same conditions. Data are presented as mean ± SD of the three independent measurements (*n* = 3).

**Figure 4:**
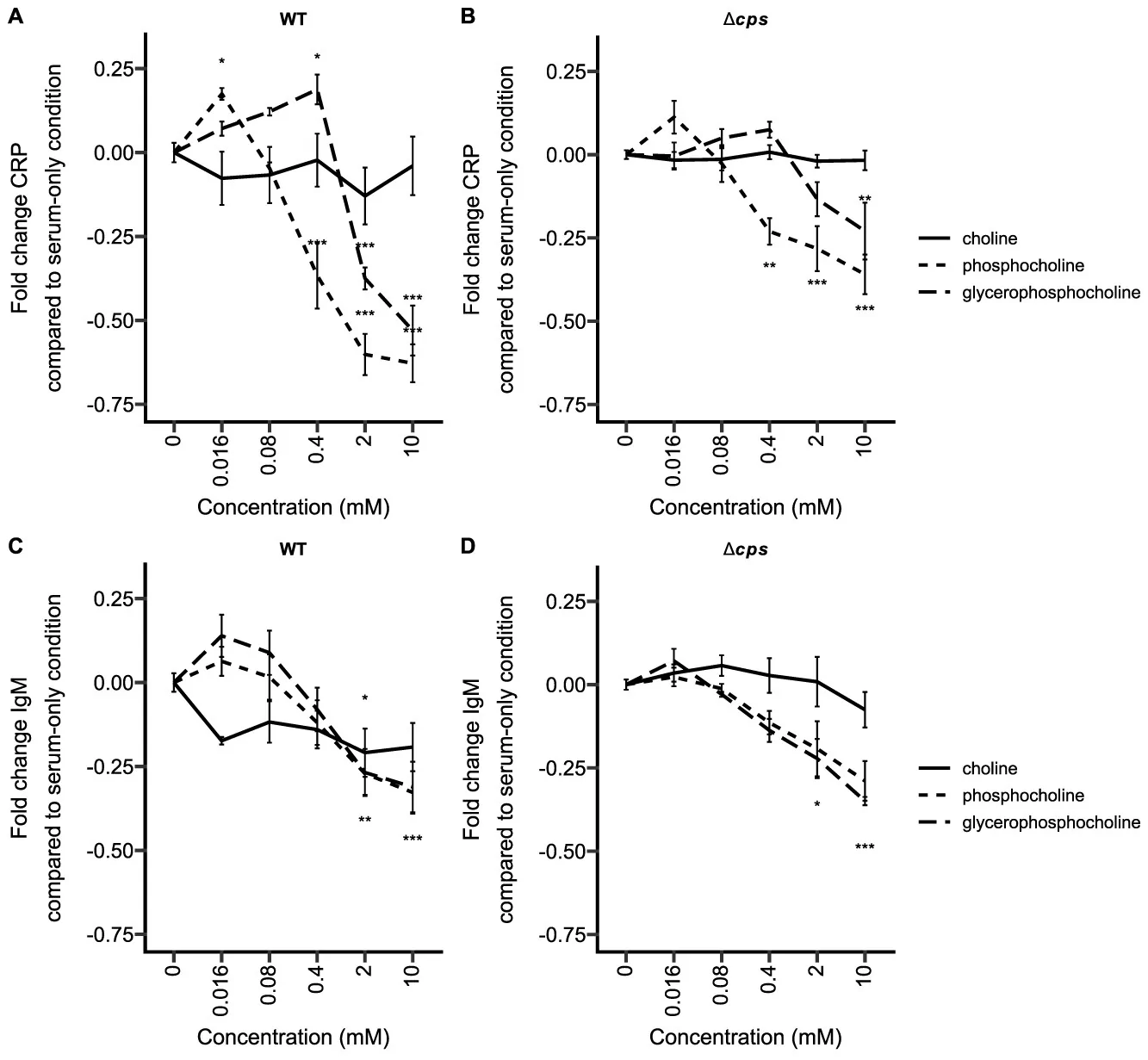
Extracellular GPC competes with the pneumococcal surface for CRP and IgM binding. Serum CRP (A and B) and IgM (D and D) binding to WT and Δ*cps* BHN418 was measured in the presence of Cho, Pcho or the extracellular metabolite GPC (*n* = 3). Data show mean ± SEM fold change relative to serum-only conditions from triplicate experiments. Asterisks indicate significant differences from the untreated (0 mM) condition based on pairwise comparisons of estimated means from linear mixed models. ^***^P < .001, ^**^P < .01, ^*^P < .05. For panels c and d, bottom asterisks indicate significant differences for both Pcho and GPC; top asterisk indicates significant difference for Cho.

Possible regulation of host responses by *S. pneumoniae* may be mediated by secreted or cell surface-bound molecules. To identify extracellular metabolites, we performed an untargeted metabolomics analysis on spent culture media from BHN100 and BHN418 grown in CDM + MS and CDM. In positive ionization mode, we observed ~1000 features which displayed significantly changed intensities in both strains after 4 h of growth (Log_2_ fold change >1 or < −1, *P-*value <.05, Fig. 3A and B). After exclusion of features with low intensity, the top 25 of increased and decreased features were manually assessed. We used m/z values to search the METLIN database [36]. This analysis identified methionine sulfoxide (MetSO), nicotinic acid (NA), and GPC with changed intensity in the supernatant of pneumococcal cultures, which we validated using pure standards of each metabolite (Fig. 3C–E; Supplementary Fig. 4a–c). Of these three, MetSO and NA displayed significantly increased intensities in both CDM + MS and CDM cultures (Fig. 3F and G), whereas GPC accumulation was specific to CDM + MS cultures (Fig. 3H). Following the same workflow in negative ionization mode, we also identified MetSO (data not shown). Given that GPC accumulation occurred exclusively in CDM + MS cultures, we further explored its potential functional relevance. GPC consists of a glycerol group attached to a Pcho. Pcho moieties are abundantly present on pneumococcal cell-wall polysaccharides, and these moieties can be bound by host CRP and IgM, which subsequently induce complement activation. Increased extracellular GPC could potentially act as a decoy for CRP/IgM binding, because previous work showed that soluble Pcho could prevent serum CRP and IgM binding to *H. influenzae* [25]. We therefore examined whether GPC could inhibit binding of CRP and IgM to pneumococci. BHN418 WT and the unencapsulated Δ*cps* strain were incubated in serum dilutions with different concentrations of Cho, Pcho, or GPC (Fig. 4A and B). CRP binding was inhibited most effectively by Pcho, but also by GPC. Inhibition was stronger for WT BHN418 compared to Δ*cps* BHN418. IgM binding was inhibited by Pcho and GPC to a similar extent for both WT and Δ*cps*. Cho only inhibited IgM binding in the WT strain, and to a lesser degree than the other metabolites. Together, these results show that extracellular GPC accumulation can interfere with CRP- and IgM-binding to pneumococci, potentially reducing downstream complement activation and immune responses.

### Upper respiratory tract conditions result in more successful epithelial colonization and reduced cytokine responses

**Figure 5:**
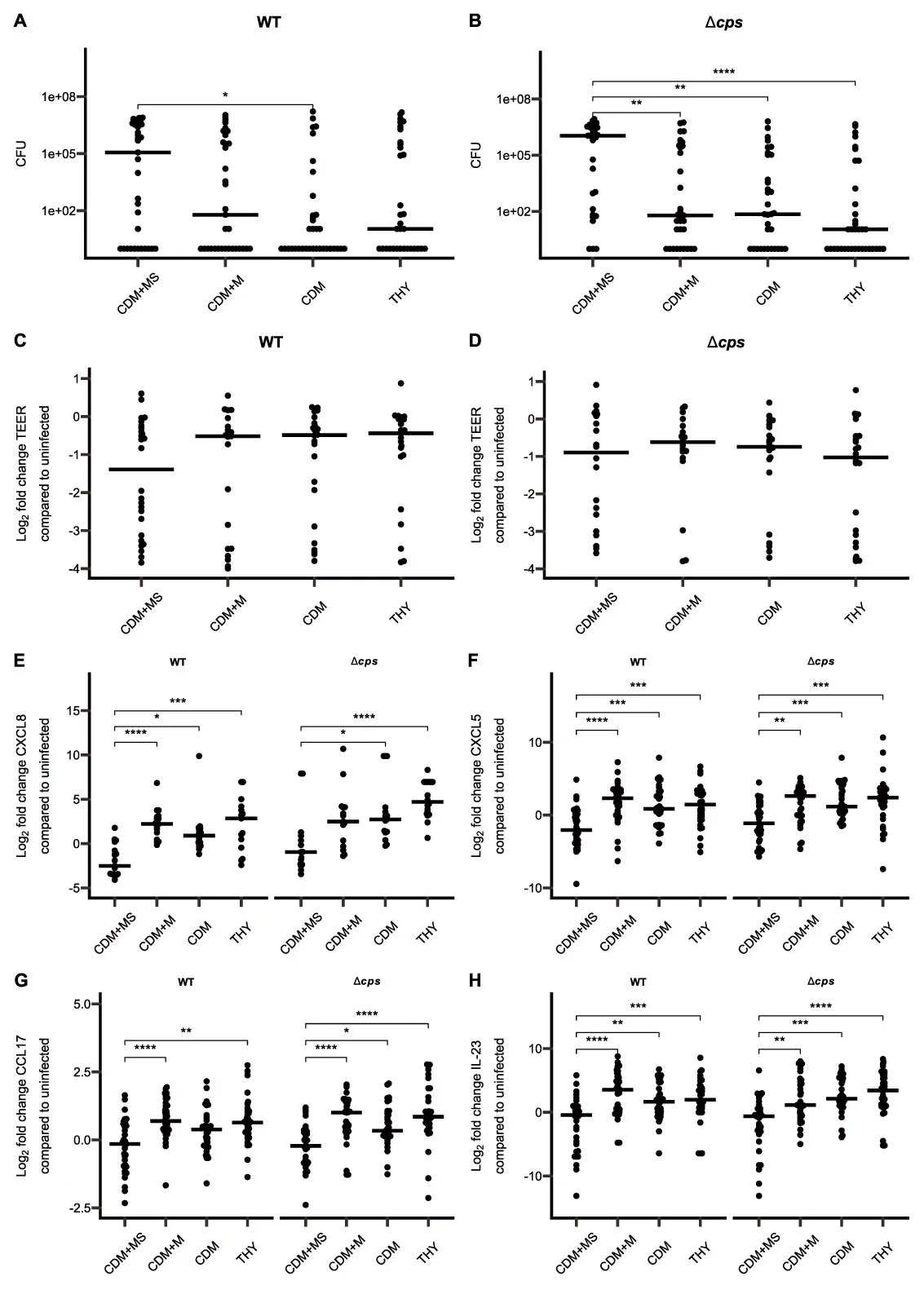
CDM + MS-grown pneumococci show enhanced colonization but induce lower cytokine responses. Effect of BHN418 growth in CDM + MS, CDM + M, CDM, and THY on subsequent infection of primary nasal epithelium inserts. CFU counts of BHN418 WT (A) and Δ*cps* (B). TEER fold change relative to uninfected epithelial layers is plotted for BHN418 WT (C) and Δ*cps* (D). Apical fold change in cytokine concentrations for CXCL8 (E), CXCL5 (F), CCL17 (G), and IL-23 (H) of all inserts shown in panels a–d compared to uninfected controls. Asterisks indicate significance from pairwise comparisons using Dunn’s test with Bonferroni multiple testing correction. ^****^P < .0001; ^***^P < .001; ^**^P < .01; ^*^P < .05. Horizontal bars indicate median. All measurements were taken 48 h after infection. For each strain and medium combination between 33 and 36 inserts were measured.

Finally, we wanted to assess whether URT conditions could also alter the interaction of pneumococci with the URT epithelium. To this end, we infected differentiated primary nasal epithelial cell layers with BHN418 WT and Δ*cps* grown in CDM + MS, CDM + M, or CDM, or the commonly used rich medium THY. Forty-eight hours post infection, CFU counts of adhered bacteria were significantly higher for BHN418 WT and Δ*cps* grown in CDM + MS compared to the other media, in which often no CFU could be detected (Fig. 5A and B). Despite the increased CFU loads in CDM + MS, TEER values, a measure of epithelial barrier status, were not significantly reduced, indicating that the higher CFU counts did not affect cell viability (Fig. 5C and D). Median TEER was only slightly lower for epithelial layers infected with CDM + MS-grown BHN418, even though median CFU load was ~1000–10 000 fold higher.

To assess epithelial cytokine responses, we measured basolateral and apical concentrations of 24 cytokines. At both 24 h and 48 h post infection, only minor differences in basolateral cytokine concentrations were observed following infection with pneumococci grown in different media (data not shown). However, apical cytokine measurements revealed a surprising reduction of CXCL8, CXCL5, CCL17, and IL-23 levels when epithelial layers were infected with CDM + MS-grown BHN418, compared to the other media, despite being exposed to higher bacterial loads (Fig. 5E–H). These results indicate that pneumococci grown in URT conditions either are less immunogenic to epithelial cells, or that they actively repress epithelial cytokine release. Exposure to the three identified metabolites did not affect cytokine concentrations in trial experiments (data not shown). Together, these results show increased pneumococcal growth on the epithelium and reduced cytokine release when pneumococci were precultured under URT conditions.

## Discussion

The widespread colonization of the human URT highlights the successful adaptation of *S. pneumoniae* to its host, which contributes to its high global disease burden. In this study, we have modeled pneumococcal adaptation to the nutrient-limited environment of the upper airways, using an *in vivo*-mimicking medium, and have demonstrated how this environment subsequently might facilitate human colonization.

Most pneumococcal growth media were developed to maximize growth, useful when identifying the causative agent of an infection [37]. However, these media do not mimic the nutrient-limited URT conditions. To investigate physiological adaptation of pneumococci to the human URT we measured monosaccharides and metal ions to closely mimic *in vivo* conditions. The observation that CDM with only monosaccharides in nasal concentrations did not support pneumococcal growth, except when mucins were supplemented, highlights the importance of this carbohydrate source in the URT. The pneumococcal genome encodes glycosidases StrH (GlcNAc), BgaA (galactose), and NanA (Neu5Ac) to release the most prevalent monosaccharides in airway mucins: GlcNAc, galactose and Neu5Ac (~20%, 20%, and 10% of mucin dry weight, respectively) [38–40]. However, not all attached monosaccharides might be accessible due to masking by undigestible components of the mucin glycan network. In fact, it has been reported that in Neu5AC-rich mucins, such as the porcine stomach mucins used in this study, resulted in the cleavage of ~40%–50% of Neu5Ac and 30%–35% of galactose and GalNAc, whereas no significant cleavage of GlcNAc was observed [41]. In nasal lavage fluid of individuals colonized by pneumococci an increase of galactose concentrations compared to negative controls was observed, as well as a NanA-dependent increase of accessible galactose on the cell surface of colonized mice [42]. Together, these results highlight the significance of galactose for pneumococci in the URT. Our observed upregulation of neuraminidase operons, galactose metabolism and sugar transporters in CDM + MS are in line with a mucin-associated galactose metabolism. This could explain the lower maximum growth within CDM + MS, because others have shown that galactose is the least preferred carbohydrate [43]. Additionally, in medium with galactose as sole carbohydrate, it has been reported that growth arrest occurred before medium galactose depletion [43].

Previously, a pneumococcal transcriptomics study using nasopharynx-mimicking conditions with galactose and Neu5Ac as carbohydrates also reported reduced growth, reduced *ply* expression, and increased galactose metabolism, compared to pneumococci grown in a glucose-rich medium [44], but no changes in expression of fatty acid metabolism and biosynthesis genes were observed. The transcriptome of pneumococci grown in their nasopharynx-mimicking medium showed some similarity to that of pneumococcal mutants lacking CcpA, an important transcriptional regulator involved in carbon catabolite repression (CCR), a system in Gram-positive bacteria regulating gene expression depending on carbohydrate availability [45]. We observed a decrease in CcpA during exponential phase, but not stationary phase. Pneumococci grown in the presence of galactose and Neu5Ac had decreased expression of the *cps*-locus and had thinner capsules [44]. They were more susceptible to opsonophagocytosis and were better colonizers of mice after intranasal challenge, compared to pneumococci grown in glucose-rich medium, but virulence in mice was greatly reduced [44].

In medium containing GlcNAc as sole carbohydrate source, pneumococci were shown to have upregulation of neuraminidase operons even though no mucin or Neu5Ac was present in the medium, suggesting expression is induced by deficiency of specific nutrients or intermediates [46]. In a transcriptome analysis comparing pneumococci grown in a medium with glucose or mucins as the sole carbohydrate source, 83 genes were upregulated when grown with mucins, of which 39 were involved in sugar processing with a clear induction of galactose metabolism and mucin-degrading pathways [43]. Out of these 83 genes, 37 (BHN100) or 41 (BHN418) showed increased protein levels during stationary phase in our experiments as well. The likely increase of saturated acids in the membrane due to downregulation of FabM and FakB3 in CDM + MS, could reduce membrane fluidity resulting in reduced mobility of proteins and possible decreased permeability [47]. We observed a decrease of FasR, a recently identified regulator of FabM and FakB3 [46], in exponential phase (BHN100 only). Whether this change in membrane composition would represent an adaptive functional response or a nonspecific stress response remains unclear.

Pneumococci colonizing the URT are mostly attached to the epithelium or growing in biofilms, not growing in a planktonic culture. This could affect gene expression profiles and local nutritional environment, and consequently pneumococcal virulence. Compared to planktonic cultures, pneumococci growing in biofilms were shown to have reduced expression of the Fab operon, *cps*-locus, *ply* and genes encoding the adhesins PcpA, PsrP, and RrgC [48], showing some similarities to our observations. Pneumococci derived from biofilm cultures did not cause bacteraemia or lethality in mice challenged intranasally, supporting the association between biofilm growth and reduced virulence [48]. Biofilm formation was shown to be dependent on carbohydrate availability: medium supplemented with monosaccharides that induce CCR, inhibited biofilm formation, whereas supplementation with galactose or lactose did not [42]. CCR affects pyruvate metabolism, a central part of pneumococcal metabolism, where it represses expression of *pfl* and *spxB*, which produce lactate and acetate, respectively. Deletion of *spxB* impairs biofilm formation, whereas increased levels of acetate promote it. In this study fatty acid metabolism was shown to be upregulated in pneumococci grown in biofilms contrasting previous observations. In the nasopharynx-mimicking medium using galactose and Neu5Ac as carbohydrates, an increased expression of both *pfl* and *spxB*, was observed [44]. In our study, we observed no differences in SpxB levels in any medium, but Pfl was increased in CDM + MS in the stationary phase, compared to both CDM and CDM + M, and compared to CDM + MS exponential phase. Levels of alcohol dehydrogenases SP_0285, SP_2026 and SP_2055, which metabolize the end products of Pfl-dependent fermentation, were similarly increased in CDM + MS [49]. SP_2026 processes acetyl-CoA, which also is a starting metabolite of the fatty acid biosynthesis pathway. The concurrent increase of SP_2026 and decrease of Fab proteins might be indicative of a change in metabolism. Taken together, these observations suggest that changes in general metabolism in different nutritional environments play an important role in biofilm formation colonization and virulence.

Changes in pneumococcal metabolism likely result in the accumulation of different metabolites. We identified three such metabolites, although many additional features were altered in CDM + MS versus CDM pneumococcal cultures, which means that there might be many other extracellular metabolites with potential effects on host–pneumococcus interactions. Additional features could not be confidently annotated due to the absence of plausible candidate metabolites, excessive numbers of potential candidates or poor signal-to-noise ratios. Nevertheless, we showed that GPC can act as a decoy of cell wall Chos for CRP and IgM binding, potentially preventing complement-mediated opsonophagocytic killing by neutrophils, an important immune response in clearing pneumococcal infections [50]. Inhibition of the complement cascade also results in lower levels of C5a and C3a, which can recruit and activate neutrophils [51]. Although concentrations of the magnitude ≥2 mM are unlikely to be achieved in planktonic cultures, GPC may accumulate locally in the mucosa reaching protective levels. Also, host enzymes could process the GPC resulting in local increases of Cho or Pcho, which could enhance (Pcho) or undo (Cho) its decoy potential, because their CRP and/or IgM binding capacity differed from GPC in our experiments [52, 53]. Further experiments are required to assess its relevance during *in vivo* infections. The observed accumulated NA in all pneumococcal CDMs is likely a product of the NAD salvage pathway. All CDMs were supplemented with niacinamide (8.2 μM), which is imported by NiaX and processed into NA, likely by PncA, and further processed to produce NAD. No differences between conditions were observed for any protein in this pathway, which corresponds with the observed NA accumulation in all conditions. Previously, deletion of PnuC, the transporter for the alternative salvageable NAD precursor, nicotinamide riboside, resulted in complete attenuation of virulence in mice [54]. Synthesis of NAD from nicotinamide riboside does not produce NA as an intermediate. The lack of virulence in Δ*pnuC* mutants could be the result of an essentiality of nicotinamide riboside in *in vivo* conditions but could also point to an important role for NA in colonization.

The observed persistent asymptomatic nasal colonization *in vivo* could be aided by preventing the induction of an epithelial cell cytokine response. Supporting this, lower cytokine induction in the nasal fluid from individuals that were successfully colonized in experimental human challenge experiments was reported previously, with a clear increase of IL-10 [55], which can induce a response of tolerance [56]. We did not observe changes in IL-10 concentration, likely because our model did not include immune cells that produce IL-10 [56]. However, our observation of lower apical concentrations of CXCL5, CXCL8, CCL17, and IL-23 during infection with pneumococci cultured under URT conditions would be in line with this hypothesis. CCL17 is an important T-cell chemokine, mostly for T_h_2 cells [57]. CXCL5 and CXLC8 are both involved in the chemotaxis and activation of neutrophils, which are crucial in battling pneumococcal infections; neutropenic mice usually succumb to pneumococcal infection [58]. Epithelial cells are the main source of CXCL5 in the mouse lung during pneumococcal infection, where less CXCL5 expression resulted in decreased neutrophil recruitment [59]. CXCL8 is one of the most abundant cytokines, produced by many cell types, including neutrophils [60]. Additionally, IL-23 is important in maintaining T cell IL-17 production, which also is involved in neutrophil recruitment [61]. A reduction in apical release of these cytokines would likely reduce the number of immune cells recruited to the URT lumen, allowing for increased pneumococcal persistence. Pneumococcal infection of epithelial cells was also shown to induce lower levels of basolateral CXCL1, CXCL5, and CXCL8 when compared to meningococcal infection [21], suggesting a pneumococcal-specific phenomenon. Although we did not observe any effect of NA during epithelial infection, NA has been shown to influence intestinal epithelial barrier, cell metabolism, and CXCL8 production when added with an inflammatory trigger [62, 63]. In addition to limiting neutrophil recruitment, resistance to neutrophil-mediated killing may be enhanced under URT conditions, because NanA, BgaA, and StrH, all upregulated in CDM + MS, also reduce C3 deposition [64]. The clear lowering of a neutrophil-chemotactic signal suggests that the URT environment creates a neutrophil avoidant pneumococcal phenotype. Whether this phenotype is a result of active repression of cytokine signaling by pneumococcal factors, or prevention of inducing an anti-pneumococcal response by masking or reducing certain immunogenic factors remains unclear. Ply can induce the release of several cytokines [19, 20, 65]. The observed lower levels of Ply in CDM + MS cultures compared to CDM and CDM + M during both growth phases could be responsible for the observed decrease in apical CXCL5, CXCL8, CCL17, and IL-23, and could suggest reduction of immunogenic factors is a key strategy for pneumococcal persistence in the URT. We only analyzed apical cytokine levels at 48 h; therefore, we could have missed short-lived responses. Neither could we examine the effect of the different growth media on the lag time during epithelial infection, nor do we know whether low CFUs were a result of autolyzed peaked population or an absence of growth. Although cytokine secretion could differ between epithelial cell donors, our experimental setup did not allow for sufficient numbers of donors to perform robust analysis of donor dependent differences.

Altogether, based on the results from this study we hypothesize that in URT, with sparse nutrient availability, the pneumococcus relies on a mucin-centered metabolism, with upregulated neuraminidases, multiple carbohydrate processing pathways and saccharide transporters, and some metal transporters. Concurrently, the pneumococcus adapts a tolerated phenotype, with decreased production of Ply and accumulation of extracellular GPC to protect against CRP and IgM. This, or other unknown repressive mechanisms also results in repression of epithelial cytokine signaling. Changes in the URT conditions occurs when barrier disruption, due to viral infections, age-related loss of function, chronic disease, or bacterial overgrowth [66–68], results in leakage of serum, rich in glucose, and other sugars into the lumen. This induces a switch in pneumococcal metabolism to more preferred carbohydrates, allowing for faster growth [43] of pneumococcal populations. Increased nutrient availability and the need for immune evasion due to increased influx of neutrophils may contribute to an increase in capsule thickness [69]. In the presence of increased numbers of neutrophils, increased levels of Ply could also prove useful because Ply can directly induce lysis of neutrophils [70], which could explain the higher levels of Ply in glucose-rich medium.

Our work demonstrates how URT nutrient conditions affect pneumococcal physiology and host interactions. The immune evasive traits, induced by limited nutrient availability, help explain how this bacterial species with substantial pathogenic potential can be carried and tolerated by a large part of the population. The marked pneumococcal differences observed in the different growth media also highlight the importance of the choice of medium in experimental design.

## Supplementary Material

Supplementary_material_wrag213

## Data Availability

Proteomics MS data and established database have been deposited to the ProteomeXchange Consortium via the PRIDE [24] partner repository with the dataset identifier PXD072730. Metabolomics MS data was deposited to the MassIVE repository with the dataset identifier MSV000100798. All other data is presented in figures and/or tables.
