# Supplementary material for: Streptococcus pneumoniae adaptation to nutrient deprivation and immune modulation drives upper respiratory tract colonization
Source: ISME J. 2026 Aug 19;20(1):wrag213. doi: 10.1093/ismejo/wrag213 (PMC13561108; doi:10.1093/ismejo/wrag213)
Supplement: Supplementary file 1 [file supplementary_material_wrag213.zip › 26-08-25 Supplementary materials.docx]

**Supplementary materials**

**Supplementary methods**

**Determination of nasal metal ion and monosaccharide concentrations**

Nasal fluid was obtained from Schirmer strips by centrifugation (10,000 g 5 min at 4°C). The obtained supernatant was adjusted to 75 µL using MilliQ. To create calibration curves for quantification in LC-MSMS, 100 µM stock solutions of D-Glucose, Galactose, N-acetyl neuraminic acid, N-acetyl-D-glucosamine, Mannose, Fucose (Supelco), Fructose, N-acetyl-D-galactosamine (Sigma Aldrich) and Maltose (BioUltra) were prepared. Each nasal fluid sample and standard was subjected to 1-phenyl-3-methyl-5-pyrazolone (PMP) derivatization as described previously [1]. Briefly, this method was down-scaled to 37.5 μL sample, 150 μL 0.2 M PMP-solution in methanol and 150 μL ammonia solution (25%). The samples were incubated for 1 h at 56°C and subsequently dried by vacuum centrifugation. The dried samples were reconstituted in MilliQ and washed 2 times with an equal volume of chloroform. Next, 20 μL of the aqueous layer from each nasal fluid sample was mixed with 20 μL of underivatized sample. A 1 μL aliquot of the mixture was directly injected to UHPLC (Agilent 1290 Infinity UHPLC) /MRM-MS (Agilent 6490 QqQ) for complete monosaccharide analysis. In between each nasal fluid sample was injected a MilliQ blank sample, to minimize carry-over. Column Type ID: Agilent ZORBAX Eclipse Plus C18 column (2.1 mm × 50 mm i.d., 1.8 μm particle size). Calibrant: Agilent ESI-TOF for mass references (m/z: 622.029, 922.010, 1221.991, 1521.971). Data analysis: Agilent MassHunter Quantitative Analysis software (version B.08). Absolute responses were determined by integration of the sugar peaks. The peaks were correctly identified based on retention time and one or two MRM transitions, similar to the literature [1]. Standard curves were prepared to quantify the peaks.

***S. pneumoniae* preculturing**

Blood agar plates were inoculated with THY-grown master stocks. After O/N growth at 37°C 5% CO_2_, colonies were collected in 1mL PBS, and used to inoculate 10 mL of preheated THY, CDM, CDM+M or CDM+MS at OD 0.05. After 4 h of incubation at 37°C 5% CO_2_, culture was aliquoted and stored at -80°C in 15% glycerol. These aliquots were then used to inoculate a second set of preheated 10 mL THY, CDM, CDM+M or CDM+MS at 1*10^6^ CFU/mL. Final stocks were obtained after 3-4 h of incubation at 37°C 5% CO­_2_ and stored at -80°C in 15% glycerol. Final stocks were used in all experiments.

**Transmission electron microscopy**

Cultures were gently pelleted (1000 × g, 5 min) and resuspended in 0.5 ml of culture medium. To this an equal volume of fixative, 2% PFA, 2.5% Glutaraldehyde, 75 mM lysine-HCl and 0.075% Ruthenium red in 0.1 M PHEM buffer (pH 6.9) (adapted from Fassel and Edmiston, 1999, method IV) [2], was added. After fixing 15 min at RT with gentle agitation, cells were pelleted (1000 × g, 5 min), resuspended in fresh fixative and fixed overnight at 4°C. After three washes with 0.1 M PHEM buffer (pH 6.9) samples were post-fixed for 1.5 h on ice with 1% OsO4 + 1.5% K_3_Fe(CN)_6_ in 0.1 M PHEM buffer (pH 6.9), followed by four washes with ultra-pure water. Subsequently samples were dehydrated on ice using a graded acetone series. After four washes with 100% acetone samples were stepwise infiltrated with Epoxy resin (composition; Sigma-Aldrich epoxy embedding medium 43%, DDSA 50%, MNA 14% and DMP-30 1.5%) and cured for 3 days at 70°C. Resin blocks were trimmed by hand and 50 nm sections were cut on a diamond knife (45° Ultra, Diatome) using a Reichert-Jung Ultracut-E ultramicrotome. Sections were collected on 100# copper grids with a carbon-coated formvar support film. Grids were post-stained with 0.5% UA and Reynolds lead citrate before analysis in a JEOL-JEM1400 Flash TEM, operating at 120 kV.

**Proteomics preparation, data acquisition and analysis**

Bacterial cells were prepared for mass spectrometry (MS) as described previously [3]. In brief, bacterial cells were disrupted in a bead mill (Retsch GmbH, Germany; 3 min, 30 Hz) and the bacterial cell powder was resuspended in 20 mM HEPES (pH 8) using four-times the volume used in the bead mill. DNA was degraded by incubating the lysates with Pierce™ Universal Nuclease (Pierce, Thermo Fisher Scientific, USA; 2.5 U, 4 mM MgCl_2_) prior to ultra-sonication for 5 min in an ultrasonic bath (Sonorex, Germany). To remove cell debris, samples were centrifuged (30 min, 17,000 x g, room temperature). Afterwards, protein concentration was determined using the Micro BCA Protein Assay Kit (Pierce, Thermo Fisher Scientific, USA) and analyzed as described [3].

Tryptic digestion of proteins and peptide purification were accomplished using an adapted single-pot solid-phase-enhanced sample preparation (SP3) protocol [4]. In brief, to each 4 µg sample protein a 1:1 mixture of hydrophilic (GE Healthcare, UK) and hydrophobic (Thermo Fisher Scientific, USA) carboxylate-modified magnetic SeraMag Speed Beads and acetonitrile (ACN) to a final concentration of 70% (v/v) were added and samples were incubated for 10 min at RT and shaking. The beads with the bound proteins were then washed twice with 70% (v/v) ethanol and once with 100% ACN at a magnetic rack. For protease digestion, the bead-bound proteins were suspended in a 25 µl of 20 mM aqueous ammonium bicarbonate containing 80 ng trypsin (Promega, USA). Samples were incubated for 16 h at 37°C prior to peptide elution with 2% (v/v) dimethyl sulfoxide and transfer to an MS vial, as described [4].

**Proteomics data processing**

MS data were analyzed using a directDIA approach in the Spectronaut software (version 18.0.230605.50606; Biognosys AG, Switzerland). A fasta-database combining the amino acid sequences of both strains was built for this project using Roary [5] and contained entries for 2377 proteins. It comprises a single representative nucleotide sequence from each protein of the two strains as pan genome (minimum percentage of the sequence identity 95%). Detailed parameters for the search are provided in Sup. Table 6.

Rarefaction curves were generated to evaluate proteomic sampling depth based on the cumulative accumulation of quantified DIA precursor features. Spectronaut report files were used as input. Precursor features were retained if they were identified and passed a *Q* value threshold of 0.01, and entries with missing precursor identifiers, protein groups, or quantitative values were removed. For each experimental condition, quantitative precursor feature values from biological replicates were aggregated by summation. Within each condition, precursor features were ranked according to their measured abundance and cumulatively accumulated from high to low abundance. Sampling depth was evaluated in incremental steps of 500 precursor features until the total number of quantified precursor features per condition was reached. At each accumulation step, the number of uniquely identified protein groups was determined. Curves approaching a plateau indicate in-creased sampling saturation. Resulting curves were generated separately for each combination of strain, cultivation medium, and growth phase. The analysis and visualization were performed in R version 4.5.3 using tidyverse (v2.0.0, https://joss.theoj.org/papers/10.21105/joss.01686), data.table (v1.18.2.1, https://rdatatable.gitlab.io/data.table/), and ggplot2 (v4.0.3; Wickham H (2016) ggplot2: Elegant Graphics for Data Analysis. Springer-Verlag New York. https://ggplot2.tidyverse.org).

The overlap of identified protein groups between experimental conditions was visualized using UpSet plots. A binary presence/absence matrix of protein group identifications generated during the published SpectroPipeR workflow [6] was used as input. Missing values were replaced by zero, and binary columns representing experimental conditions were retained for analysis. UpSet plots were generated using the UpSetR package (v1.4.0, https://doi.org/10.1093/bioinformatics/btx364) based on the published SpectroPipeR workflow. Protein group intersections were visualized across the twelve experimental conditions representing combinations of strain, cultivation medium, and growth phase. Intersections were ranked according to frequency, and empty intersections were omitted. The main bar plot displays the number of shared protein groups between conditions, whereas set size bars indicate the number of protein groups identified per condition.

Globally median normalized data processed with Spectronaut were further evaluated using R (v4.1.3) via an in-house version of the recently published SpectroPipeR workflow, employing the same R packages [6]. The key analysis parameters were as follows: Peptides containing oxidized methionine were excluded from the dataset before performing any quantitative analysis. For global proteome profiling, peptide ions were retained only if they were confidently identified (*Q*- value ≤ 0.001) in at least 50% of one experimental condition. Peptide intensities were determined by summing up intensities of all corresponding ions. Peptide-level ROPECA statistics [7] were computed on a per-protein basis for individual condition comparisons, followed by *P* value correction using the Benjamini-Hochberg method. Only protein groups identified with at least two distinct peptides were included in statistical analysis and subsequent protein-level analyses. Non-unique protein groups were excluded from the dataset. Protein abundance estimates were derived from the normalized peptide intensities using the maxLFQ algorithm [8].

PCoA and PCA were performed using maxLFQ abundance estimates of all resulting proteins. The volcano plots show the results of peptide-level ROPECA statistics[7]. These were also used to determine whether protein levels differed between conditions: a log2 ratio of <-1 or >1 with adjusted *P* value <0.05. Sequences of proteins that differed in specific media comparisons were used for clustering and GO-term analysis using STRING-database [9]. Additionally, PneumoBrowse2 [10] was used to examine possible associated (predicted) operons based on transcription start- and termination sites.

**Exometabolome mass spectrometry**

Liquid chromatography–mass spectrometry analyses were performed on an Agilent 1290 II LC system coupled to an Agilent 6546 high-resolution quadrupole time-of-flight mass spectrometer as described [11]. In brief, a 2 µL sample was injected onto a Diamond Hydride type C column (150 X 2.1 mm, 4 µm) and separated using a 0.4 mL/min gradient of water with 0.2% formic acid in acetonitrile with 0.2% formic acid (B) (0–2 min, 85% B; 3–5 min, 80% B; 6–7 min, 75% B; 8–9 min, 70% B; 10–11 min, 50% B; 11–14 min, 20% B; 14–24 min, 5% B; followed by 10 min re-equilibration at 85% B). Mass spectra were recorded from m/z 50–1200 in the positive and negative ionization mode. The resulting data were converted to mzXML format using proteowizard software [12] and analyzed using XCMS online [13].

Fragmentation spectra of reference MetSO, GPC and NA (at 10 µM), and the differentially present features were obtained using the LC-MS setup described above and isolating the corresponding masses (166.1, 258.1, and 124.0 [M+H]^+^, respectively) at narrow quadrupole isolation width and collision energies of 10, 20, and 40 V. Data were recorded and analyzed using Agilent Masshunter Workstation LC/MS Acquisition (version 10.1) and Qualitative Analysis (version 10.0), respectively.

For both positive and negative ionization mode, change in feature intensity after 4 h of pneumococcal growth was assessed by calculating log2-fold change and performing Welch’s t-test. Before further examination of the data, the features that met the following criteria were removed: only observed in the blanks, a *P* value of > 0.05, an intensity of <1*10^5^ at t = 0 h for features with negative fold change, an intensity of <1*10^5^ at t = 4 h for features with positive fold change. Then, only the features that changed in both pneumococcal strains were ranked in fold change, and the top 25 of decreased and increased features were attempted to manually identify, based on available data.

**Epithelial cell culture**

Epithelial culture methods have been described previously in [14, 15]. In short, healthy post-operative residual tissues was collected from three individuals. Collected tissue was treated with antibiotics, brought into suspension using DNAse and collagenase, cultured in expansion medium (supplemented PneumaCult-Ex Basal medium; Stemcell Technologies), and stored at -135°C. T-75 flasks containing expansion medium, consisting of PneumaCult-Ex Plus Medium (StemCell) supplemented with PneumaCult-Ex Plus Supplement 50× (StemCell), Hydrocortisone Stock Solution 200× (StemCell) and penicillin/streptomycin 100× (Sigma-Aldrich), were inoculated with 1*10^6^ cells from frozen stocks, and incubated at 37°C in 5% CO_2_. Medium was replaced every other day until 90% confluency was reached. 0.4 µm inserts of 24-wells plates were coated with collagen type I. Cells in confluent T-75 flasks were resuspended using trypsin and 1*10^5^ cells in 100 µL of expansion medium was added to the apical side of the insert. 500 µL of expansion medium was added to the basolateral side and plates were incubated at 37°C in 5% CO_2_. Medium was replaced every other day until confluent, at which point the apical medium was removed, and basolateral medium replaced with differentiation medium consisting of PneumaCult-ALI basal medium (StemCell) supplemented with PneumaCult-ALI supplement 10× (StemCell), PneumaCult-ALI maintenance supplement 100× (StemCell), Hydrocortisone Stock Solution 200× (StemCell), Penicillin-Streptomycin 100× (P/S, Sigma-Aldrich) and heparin 500× (StemCell). Now exposed to air apically, the cell layer was left to differentiate for 4-6 weeks at 37°C in 5% CO_2_, with differentiation medium replacement three times a week. From week 2-3, the apical side was washed weekly with PBS to remove mucus buildup.

**Epithelial cytokine measurements**

Cytokine concentrations were determined using two LEGENDplex kits (Human Proinflammatory Chemokine Panel 1-740985and Human Inflammation Panel 1-740809) following manufacturer’s protocol. Included cytokines are CXCL1, CXCL5, CXCL8, CXCL9, CXCL10, CXCL11, CCL2, CCL3, CCL4, CCL5, CCL17, CCL20, IL-1β, IL-6, IL-10, IL-12p70, IL-17A, IL-18, IL-23, IL-33, IFNα2, IFNγ and TNFα. Apical cytokine measurements using the Human Proinflammatory Chemokine Panel 1 for half of the inserts were done on undiluted samples and for the other half on 1:5 dilution of samples. For Fig. 6, fold change in concentration of each cytokine compared to the median concentration of uninfected samples of correlating medium was calculated. As an example, apical CXCL8 concentrations in CDM-grown BHN418 WT infected inserts, were compared to apical median CXCL8 concentration in mock 1:10 CDM infection. CXCL8 measurements in undiluted apical samples were very close to the upper detection limit and therefore excluded from the results. Fig 6E therefore represents the data of the remaining 15-18 inserts per condition.

**Data analysis and statistics**

All R-scripts used to create the main figures are included as a supplement. Unless otherwise mentioned, analysis and visualization were done using R version 4.6.1. To analyze differences in the growth curves, we fitted a linear mixed-effects model using the lmer function of the lme4 package (v2.0-6), with medium, time, and strain as fixed effects and experiment as a random intercept. Estimated marginal means (EMMs) were computed using the emmeans package (v2.0.4), and pairwise comparisons between media were performed for each strain and time point, with Tukey adjustment for multiple testing. Area under the curve (AUC) was calculated for each experiment, medium, and strain using the trapezoidal rule on log-transformed CFU data. A linear mixed-effects model was fitted to the AUC values with medium as fixed effect and experiment as a random intercept. Strain exclusion resulted in better AIC and BIC. EMMs and pairwise comparisons between media were calculated and *P* values adjusted for multiple comparisons using Tukey’s method.

For proteomics PCoA, missing values were imputed as zero, Bray-Curtis dissimilarity was calculated using the vegan package (v2.7-5) and PCoA was performed with cmdscale. For PCA, data were centered and scaled, and factoMineR (v2.16) and factoextra (v2.2.0) were used. ROPECA statistics [7] uses peptide measurements to determine protein-level changes. Using bootstrap sampling, it maximizes overlap of top-ranked features across resampled data for feature selection. Then, all available peptide-level data are used to calculate peptide intensity ratios between conditions, and the significance of median p-values is estimated using the beta-distribution. The ComplexHeatmap package (v2.28.0) was used for the proteomics heatmap. GO-term analysis of the pneumococcal proteome was performed using the STRINGdb package (v2.24.0), version 12.0, with a minimum required confidence score for interactions of 200, including all types of evidence for protein-protein interactions, using all available linking data.

For figure 5, a linear mixed-effects model, with cholines in different concentrations, and strain as fixed effects and experiment as a random intercept. EMMs were calculated for each treatment and compared to the serum only condition, with Sidak adjustment for multiple testing.

**Sup. Fig. 1: Continuation of Figure 1, growth in the different media.** (A) No pneumococcal growth in CDM+M with *in* vivo nasal monosaccharide concentrations (CDM+MS without mucin supplementation). Increasing monosaccharide concentrations allowed growth. CDM+MS contains 1x *in vivo*-monosaccharide concentrations with mucin supplementation. (B) Area under the curve (AUC) calculation of each measured CFU growth curve of both strains in CDM, CDM+M and CDM+MS with mucin as shown in Fig. 2A and B. Estimated means with 95%-confidence interval of linear mixed model shown in brown. Asterisks indicate significant pairwise differences between media using model-based comparisons with Tukey’s correction (** *P* < 0.01). Colony forming units (CFU/mL; C, circles), flow cytometry event counts (C, triangles) and OD_620nm_ measurements (D) of a subset of BHN418 growth curves also shown in figure 2 for which the other measurements were performed. Colors represent the specific replicate experiment. No OD_620nm_ measurements of CDM+MS were taken due to presence of mucins in the medium that raised the OD_620nm_ to such a degree that bacterial growth could not be measured by change in OD_620nm_.

**Sup. Fig. 1 alt-text:** Graphs labelled A to D showing additional growth data of BHN100 and BHN418 in the different media. A depicts growth curves in CDM+MS without mucin. Line types indicate the different monosaccharide concentrations. B shows area under the curve calculations of individual growth curves, with the estimated mean and 95% confidence interval plotted. C and D show alternative measurements of pneumococcal growth, OD and measured flow cytometry events, which show comparable levels or patterns to the CFU measurement as shown in Figure 1.

**Sup. Fig. 2: Assessment of proteome sampling depth and protein group recovery across the different conditions of the DIA-proteomics data as in Fig. 2.** (A) Rarefaction curves showing the cumulative number of unique protein groups plotted against the accumulated quantified precursor features. Quantified precursor features were accumulated from highest to lowest abundance. Curves approaching a plateau indicate increased sampling saturation. Growth medium (colors), growth phase (linetype) and strain (shape) of conditions are indicated. (B) UpSet plot visualizing the number of shared proteins across conditions. The main bar plot displays the number of shared protein groups between conditions, whereas set size bars indicate the number of protein groups identified per condition. Intersections were ranked according to frequency, and empty intersections were omitted.

**Sup. Fig. 2 alt-text:** Graphs A and B depicting proteomics sampling depth and differences between samples. A shows rarefaction curves showing accumulated quantified precursor features of each condition (indicated by color and shape) to assess sampling depth. B is an upSet plot that shows the number of proteins detected that are shared between conditions. The top part shows the number, the bottom shows for which combination of conditions these proteins were shared. On the left, the total number of unique proteins is indicated per condition.

**Sup. Fig. 3: Continuation of Fig. 2: additional plots of pneumococcal proteome profiles in the different media.** (A) Principal analysis of proteomes in different growth media. Dot color and shape indicate, growth medium, pneumococcal strain and growth phase. GO-enrichment analysis of proteins that significantly differed between CDM-grown and CDM+MS-grown BHN100 and BHN418. False discovery rates (FDR) of Fischer’s exact test with Benjamini-Hochberg are shown for the top 15 GO biological process terms (B) and KEGG pathways (C).

**Sup. Fig 3 alt-text:** Graphs labelled A to C that offer additional proteomics analysis. A shows the results of PCA analysis with conditions indicated by color and shape. B and C show bar plots indicating the GO biological processes and KEGG pathways of which proteins were significantly differentially produced.

**Sup. Table 1: Increased or decreased proteins assigned to protein groups as shown in figure 2.** Names derived from TIGR4 protein names, or R6 when no related-TIGR4 gene was detected.

**Sup. Table 2: Proteins associated with each GO-process as shown in Sup. Fig. 3.** Colors indicate upregulation (yellow) or downregulation (blue). Names derived from R6 reference strain.

**Sup. Table 3: Proteins associated with each KEGG pathway as shown in Sup. Fig. 3.** Colors indicate upregulation (yellow) or downregulation (blue). Names derived from R6 reference strain.

**Sup. Fig. 4: Continuation of Fig. 3.** Extracted ion chromatograms for m/z values: 166.0537 (MetSO; panel A), 124.0399 (NA; panel B), 258.1110 (GPC; panel C). Blue: standard solutions of individual metabolites. Black: t = 4 h experimental sample.

**Sup. Fig. 4 alt-text:** Extracted ion chromatograms of identified metabolites with a reference sample in blue and an experimental sample in black.

**Materials and methods supplements**

**Sup. Table 4: Chemically defined media components concentrations**

| Component | CDM base | | |
| --- | --- | --- | --- |
| β-glycerophosphate disodium salt hydrate | 105 mM | | |
| Potassium dihydrogen phosphate | 7.3 mM | | |
| ammonium citrate tribasic | 2.5 mM | | |
| Sodium acetate | 12.2 mM | | |
| Pyridoxal hydrochloride | 9.8 µM | | |
| Thiamine hydrochloride | 3.0 µM | | |
| Riboflavin | 2.7 µM | | |
| Calcium pantothenate | 4.2 µM | | |
| Biotin | 0.4 µM | | |
| Folic acid | 2.3 µM | | |
| Niacinamide | 8.2 µM | | |
| L-alanine | 2.7 mM | | |
| L-glutamine | 2.7 mM | | |
| L-asparagine monohydrate | 2.7 mM | | |
| L-arginine | 0.7 mM | | |
| L-lysine monohydrochloride | 2.4 mM | | |
| L-isoleucine | 1.6 mM | | |
| L-methionine | 0.8 mM | | |
| L-phenylalanine | 1.7 mM | | |
| L-serine | 3.2 mM | | |
| L-threonine | 1.9 mM | | |
| L-tryptophane | 0.2 mM | | |
| L-valine | 2.8 mM | | |
| Glycine | 2.3 mM | | |
| L-histidine | 0.7 mM | | |
| L-leucine | 3.6 mM | | |
| DL-proline | 0.6 mM | | |
| L-cysteine | 1.7 mM | | |
| L-aspartic acid | 2.3 mM | | |
| Choline chloride | 36 µM | | |
| Adenine | 74 µM | | |
| Uracil | 89 µM | | |
| Sodium pyruvate | 91 µM | | |
|  |  | | |
|  | CDM | CDM+M | CDM+MS |
| Magnesium chloride hexahydrate | 1.0 mM | - | - |
| Calcium chloride | 0.3 mM | - | - |
| Zinc sulfide heptahydrate | 17 µM | - | - |
| Cobalt chloride hexahydrate | 11 µM | - | - |
| Copper(II) sulfate pentahydrate | 0.4 µM | - | - |
| Manganese (II) sulfate monohydrate | 166 µM | - | - |
| Manganese standard | - | 0.7 µM | 0.7 µM |
| Magnesium standard | - | 1.4 mM | 1.4 mM |
| Calcium standard | - | 1.7 mM | 1.7 mM |
| Cobalt standard | - | 0.7 µM | 0.7 µM |
| Copper standard | - | 6.5 µM | 6.5 µM |
| Zinc standard | - | 12 µM | 12 µM |
| Iron standard | - | 21 µM | 21 µM |
| glucose | 28 mM | 28 mM | 65 µM |
| fucose | - | - | 0.3 µM |
| GlcNAc | - | - | 0.03 µM |
| GalNAc | - | - | 5 µM |
| fructose | - | - | 0.3 µM |
| mannose | - | - | 0.2 µM |
| galactose | - | - | 0.05 µM |
| maltose | - | - | 11 µM |
| Neu5Ac | - | - | 12 µM |
| mucin from porcine stomach | - | - | 0.1 g/L |

**Sup. Table 5: nanoLC-MS/MS Settings**

| **Reversed phase liquid chromatography (RPLC)** | |  |
| --- | --- | --- |
| Instrument | Ultimate 3000 RSLC (Thermo Scientific) |  |
| Trap column | 75 μm inner diameter, packed with 3 μm C18 particles (Acclaim PepMap100, Thermo Scientific) |  |
| Analytical column | Accucore 150-C18, (Thermo Fisher Scientific)  25 cm x 75 μm, 2.6 μm C18 particles, 150 Å pore size |  |
| Buffer system | Binary buffer system consisting of 0.1% acetic acid in HPLC-grade water (solvent A) and 100% ACN in 0.1% acetic acid (solvent B) |  |
| Flow rate | 300 nL/min |  |
| Gradient | Linear gradient of solvent B from 2% up to 25% for intracellular proteome analysis |  |
|  | 0min-2%  2min-5%  10min-7%  70min-25%  75min-40%  77min-90%  83min-90%  85min-2%  95min-2%  gradient of solvent B for secretome analysis |  |
| Gradient duration | 120 min |  |
| Column oven temperature | 40°C |  |
| **MS/MS Intracellular proteome** | | |
| Instrument | Q Exactive HF mass spectrometer (Thermo Fisher Scientific) | |
| Electrospray | Nanospray Flex™ Ion Source | |
| Operation mode | Data-independent | |
| **Full scan properties** |  | |
| MS scan resolution | 60000 | |
| Normalized AGC target | 5e6 | |
| Maximum ion injection time for the MS scan | 200 ms | |
| Scan range | 333 to 1650 m/z | |
| RF Lens | 50 % | |
| Spectra data type | Profile | |
| **Dia properties (MS2)** |  | |
| Precursor mass range | 333 to 1650 m/z | |
| Resolution | 30,000 | |
| Norm. MS/MS AGC target | 3e6 | |
| Maximum ion injection time mode | Auto | |
| Spectra data type | Profile | |
| Microscans | 1 | |
| Isolation window | 56 windows, 13 m/z, 2 m/z overlap | |
| Define first mass | 200 | |
| Dissociation mode | Higher energy collisional dissociation (HCD) | |
| Normalized collision energy | 27.5 % | |
| **MS/MS Secretome** | | |
| Instrument | Orbitrap Exploris 480 mass spectrometer (Thermo Fisher Scientific) | |
| Electrospray | Nanospray Flex™ Ion Source | |
| Operation mode | Data-independent | |
| **Full scan properties** |  | |
| MS scan resolution | 120000 | |
| AGC target | 3e6 (300%) | |
| Maximum ion injection time for the MS scan | 60 ms | |
| Scan range | 350 to 1200 m/z | |
| Microscans | 1 | |
| Polarity | Positive | |
| RF lens | 50% | |
| Spectra data type | Profile | |
| **Dia properties (MS2)** |  | |
| Resolution | 30000 | |
| Maximum ion injection time for the MS/MS scans | Auto | |
| Normalized AGC target | 3e6 | |
| Spectra data type | Profile | |
| Microscans | 1 | |
| Isolation window | 66 | |
| Isolation window width | 13 m/z | |
| Window overlay | 2 m/z | |
| Fixed first mass | 200 | |
| HCD collision energy | 30% | |

**Sup. Table 6: Spectronaut database search settings:**

Spectronaut 18.0.230605.50606

Analysis Type: directDIA

Analysis Date: 05-July-2023 21:15:32 UTC+0

[BEGIN-SETTINGS]

Settings Used: C_FunGene_directDIA_sparse_no_imputing

├─ DIA Analysis\Calibration

│ ├─ MZ Extraction Strategy:	Maximum Intensity

│ ├─ Allow source specific iRT Calibration:	True

│ ├─ Precision iRT:	True

│ │ ├─ Exclude De-amidated Peptides:	True

│ │ └─ iRT <-> RT Regression Type:	Local (Non-Linear) Regression

│ ├─ MS1 Mass Tolerance Strategy:	System Default

│ └─ MS2 Mass Tolerance Strategy:	System Default

├─ DIA Analysis\Identification

│ ├─ Precursor Qvalue Cutoff:	0.001

│ ├─ Precursor PEP Cutoff:	0.2

│ ├─ Protein Qvalue Cutoff (Experiment):	0.01

│ ├─ Protein Qvalue Cutoff (Run):	0.05

│ ├─ Protein PEP Cutoff:	0.75

│ ├─ Single Hit Definition:	By Stripped Sequence

│ ├─ Exclude Single Hit Proteins:	False

│ ├─ Exclude Duplicate Assays:	True

│ ├─ Exclude Predicted Fragment Scores:	False

│ ├─ Generate Decoys:	True

│ │ ├─ Decoy Generation Method:	Mutated

│ │ │ └─ Preferred Fragment Source:	NN Predicted Fragments

│ │ └─ Decoy Limit Strategy:	Dynamic

│ │	└─ Library Size Fraction:	0.1

│ └─ Pvalue Estimator:	Kernel Density Estimator

├─ DIA Analysis\Pipeline Mode

│ ├─ Generate SNE File:	True

│ │ └─ Store Ion traces in SNE:	False

│ ├─ Post Analysis Reports:

│ │ ├─ CV Density Line Chart:	True

│ │ ├─ CVs Below X Bar Chart:	True

│ │ ├─ Data Completeness Bar Chart:	True

│ │ ├─ Run Identifications Bar Chart:	True

│ │ └─ Scoring Histograms:	True

│ ├─ Report Schema:	C_FunGene_complex (Normal)

│ └─ Reporting Unit:	Across Experiment

├─ DIA Analysis\Post Analysis

│ ├─ Differential Abundance Testing:	Paired t-test

│ │ ├─ Group-Wise Testing Correction:	False

│ │ ├─ Log2 Ratio Candidate Filter:	0.58

│ │ └─ Confidence Candidate Filter:	Qvalue

│ │	└─ Confidence:	0.05

│ ├─ Differential Abundance Grouping:	Major Group (Quantification Settings)

│ │ └─ Smallest Quantitative Unit:	Precursor Ion (Quantification Settings)

│ │	└─ Use All MS-Level Quantities:	False

│ ├─ Calculate Explained TIC:	None

│ ├─ Calculate Sample Correlation Matrix:	True

│ └─ Hierarchical Clustering:	True

│	├─ Distance Metric:	Manhattan Distance

│	├─ Linkage Strategy:	Ward's Method

│	├─ Order Runs by Clustering:	True

│	└─ Z-score Transformation:	False

├─ DIA Analysis\Protein Inference

│ └─ Protein Inference Workflow:	Automatic

│	└─ Inference Algorithm:	IDPicker

├─ DIA Analysis\PTM Workflow

│ └─ PTM Localization:	False

├─ DIA Analysis\Quantification

│ ├─ Precursor Filtering:	Identified (Qvalue)

│ │ └─ Imputation Strategy:	Use Background Signal

│ ├─ Proteotypicity Filter:	None

│ ├─ Protein LFQ Method:	MaxLFQ

│ ├─ Quantity MS Level:	MS2

│ ├─ Quantity Type:	Area

│ ├─ Cross-Run Normalization:	True

│ │ ├─ Normalization Filter Type:	None

│ │ ├─ Normalization Strategy:	Local Normalization

│ │ └─ Row Selection:	Identified in at least 1 Run (Sparse)

│ ├─ Quantification window:	Not Synchronized (SN 17)

│ ├─ Interference Correction:	True

│ │ ├─ Only Identified Peptides:	True

│ │ ├─ Exclude All Multi-Channel Interferences:	True

│ │ ├─ MS1 Min:	2

│ │ └─ MS2 Min:	3

│ ├─ Major (Protein) Grouping:	by Protein Group Id

│ ├─ Minor (Peptide) Grouping:	by Stripped Sequence

│ ├─ Major Group Quantity:	Mean peptide quantity

│ ├─ Major Group Top N:	True

│ │ ├─ Max:	3

│ │ └─ Min:	2

│ ├─ Minor Group Quantity:	Sum precursor quantity

│ └─ Minor Group Top N:	False

├─ DIA Analysis\Workflow

│ ├─ Method Evaluation:	False

│ ├─ MS2 DeMultiplexing:	Automatic

│ ├─ Profiling Strategy:	iRT Profiling

│ │ ├─ Carry-over exact Peak Boundaries:	False

│ │ ├─ Profiling Row Selection:	Minimum Qvalue Row Selection

│ │ │ └─ Qvalue Threshold:	0.001

│ │ └─ Profiling Target Selection:	Profile only non-identified Precursors

│ │	├─ Identification Criterion:	Qvalue

│ │	└─ Threshold:	0.001

│ ├─ Run Limit for directDIA Library:	-1

│ └─ Unify Peptide Peaks Strategy:	Select corresponding Peak

├─ DIA Analysis\XIC Extraction

│ ├─ XIC IM Extraction Window:	Dynamic

│ │ └─ Correction Factor:	1

│ ├─ XIC RT Extraction Window:	Dynamic

│ │ └─ Correction Factor:	1

│ ├─ MS1 Mass Tolerance Strategy:	Dynamic

│ │ └─ Correction Factor:	1

│ └─ MS2 Mass Tolerance Strategy:	Dynamic

│	└─ Correction Factor:	1

├─ Pulsar Search\Identification

│ ├─ PSM FDR:	0.01

│ ├─ Peptide FDR:	0.01

│ ├─ Protein Group FDR:	0.01

│ ├─ directDIA Workflow:	directDIA+ (Deep)

│ └─ PTM Localization Filter:	False

├─ Pulsar Search\Labeling

│ └─ Channels:

│	├─ Channel 1:	False

│	├─ Channel 2:	False

│	└─ Channel 3:	False

├─ Pulsar Search\Modifications

│ ├─ Max Variable Modifications:	5

│ └─ Select Modifications:

│	├─ Fixed Modifications:

│	└─ Variable Modifications:	Oxidation (M)

├─ Pulsar Search\Peptides

│ ├─ Enzymes / Cleavage Rules:	Trypsin/P

│ ├─ Digest Type:	Specific

│ ├─ Max Peptide Length:	52

│ ├─ Min Peptide Length:	7

│ ├─ Missed Cleavages:	2

│ └─ Toggle N-terminal M:	True

├─ Pulsar Search\Result Filters

│ ├─ Fragment Ions:

│ │ ├─ Ion AA Length:	True

│ │ │ └─ N:	3

│ │ ├─ Ion Charge:	False

│ │ ├─ Ion Loss Type:	False

│ │ ├─ Ion Type:	False

│ │ ├─ m/z :	True

│ │ │ ├─ Max:	1800

│ │ │ └─ Min:	300

│ │ └─ Relative Intensity:	True

│ │	└─ Min:	5

│ └─ Precursors:

│	├─ Amino Acids:	False

│	├─ Best N Fragments per Peptide:	True

│	│ ├─ Max:	10

│	│ └─ Min:	6

│	├─ Best N Peptides per Protein Group:	False

│	├─ Channel Count:	False

│	├─ FASTA Matched:	False

│	├─ Missed Cleavage:	False

│	├─ Modifications:	None

│	├─ Peptide Charge:	False

│	└─ Proteotypicity:	False

├─ Pulsar Search\Speed-Up

│ ├─ IM DFD Processing:

│ │ └─ Use Dynamic IM Peak Filter:	True

│ │	└─ Target TIC Fraction:	0.9

│ └─ MS2 Index:	Automatic

├─ Pulsar Search\Tolerances

│ └─ Tolerance Parameters:

│	├─ Thermo IonTrap:

│	│ ├─ Calibration Search:	Dynamic

│	│ │ ├─ MS1 Correction Factor:	1

│	│ │ └─ MS2 Correction Factor:	1

│	│ └─ Main Search:	Dynamic

│	│	├─ MS1 Correction Factor:	1

│	│	└─ MS2 Correction Factor:	1

│	├─ Thermo Orbitrap:

│	│ ├─ Calibration Search:	Dynamic

│	│ │ ├─ MS1 Correction Factor:	1

│	│ │ └─ MS2 Correction Factor:	1

│	│ └─ Main Search:	Dynamic

│	│	├─ MS1 Correction Factor:	1

│	│	└─ MS2 Correction Factor:	1

│	└─ TOF:

│	├─ Calibration Search:	Dynamic

│	│ ├─ MS1 Correction Factor:	1

│	│ └─ MS2 Correction Factor:	1

│	└─ Main Search:	Dynamic

│	├─ MS1 Correction Factor:	1

│	└─ MS2 Correction Factor:	1

└─ Pulsar Search\Workflow

├─ Fragment Ion Selection Strategy:	Intensity Based

├─ In-Silico Generate Missing Channels:	False

└─ Use DNN Predicted Ion Mobility:	Auto

[END-SETTINGS]

**References**

1.	Xu, G., et al., *Revisiting monosaccharide analysis - quantitation of a comprehensive set of monosaccharides using dynamic multiple reaction monitoring.* Analyst, 2017. **143**(1): p. 200–207.

2.	Fassel, T.A. and C.E. Edmiston, Jr., *Ruthenium red and the bacterial glycocalyx.* Biotech Histochem, 1999. **74**(4): p. 194–212.

3.	Reder, A., et al., *MassSpecPreppy-An end-to-end solution for automated protein concentration determination and flexible sample digestion for proteomics applications.* Proteomics, 2024. **24**(9): p. e2300294.

4.	Blankenburg, S., et al., *Improving proteome coverage for small sample amounts: an advanced method for proteomics approaches with low bacterial cell numbers.* Proteomics, 2019. **19**(23): p. e1900192.

5.	Page, A.J., et al., *Roary: rapid large-scale prokaryote pan genome analysis.* Bioinformatics, 2015. **31**(22): p. 3691–3.

6.	Michalik, S., et al., *SpectroPipeR-a streamlining post Spectronaut DIA-MS data analysis R package.* Bioinformatics, 2025. **41**(3).

7.	Suomi, T. and L.L. Elo, *Enhanced differential expression statistics for data-independent acquisition proteomics.* Sci Rep, 2017. **7**(1): p. 5869.

8.	Cox, J., et al., *Accurate proteome-wide label-free quantification by delayed normalization and maximal peptide ratio extraction, termed MaxLFQ.* Mol Cell Proteomics, 2014. **13**(9): p. 2513–26.

9.	Szklarczyk, D., et al., *The STRING database in 2023: protein-protein association networks and functional enrichment analyses for any sequenced genome of interest.* Nucleic Acids Res, 2023. **51**(D1): p. D638–d646.

10.	Janssen, A.B., et al., *PneumoBrowse 2: an integrated visual platform for curated genome annotation and multiomics data analysis of Streptococcus pneumoniae.* Nucleic Acids Res, 2024. **53**(D1): p. D839–D851.

11.	Jansen, R.S., et al., *Aspartate aminotransferase Rv3722c governs aspartate-dependent nitrogen metabolism in Mycobacterium tuberculosis.* Nat Commun, 2020. **11**(1): p. 1960.

12.	Chambers, M.C., et al., *A cross-platform toolkit for mass spectrometry and proteomics.* Nat Biotechnol, 2012. **30**(10): p. 918–20.

13.	Tautenhahn, R., et al., *XCMS Online: a web-based platform to process untargeted metabolomic data.* Anal Chem, 2012. **84**(11): p. 5035–9.

14.	van Woudenbergh, E., et al., *Primary human nasal, nasopharyngeal, and bronchial epithelia show distinct immune responses to various pathogens.* Pathog Dis, 2025. **83**: p. ftaf011.

15.	Arends, D.W., et al., *Impact of host factors and invasive meningococci on bacterial adhesion, proliferation, primary nasal epithelial barrier function, and immune response.* Microbiol Spectr, 2025. **13**(8): p. e0014125.
