# Supplementary material for: Streptococcus pneumoniae adaptation to nutrient deprivation and immune modulation drives upper respiratory tract colonization
Source: ISME J. 2026 Aug 19;20(1):wrag213. doi: 10.1093/ismejo/wrag213 (PMC13561108; doi:10.1093/ismejo/wrag213)
Supplement: Supplementary file 1 [file supplementary_material_wrag213.zip › 26-08-25 Supplementary Table 4.docx]

**Sup. Table 4: Chemically defined media components concentrations**

| Component | CDM base | | |
| --- | --- | --- | --- |
| β-glycerophosphate disodium salt hydrate | 105 mM | | |
| Potassium dihydrogen phosphate | 7.3 mM | | |
| ammonium citrate tribasic | 2.5 mM | | |
| Sodium acetate | 12.2 mM | | |
| Pyridoxal hydrochloride | 9.8 µM | | |
| Thiamine hydrochloride | 3.0 µM | | |
| Riboflavin | 2.7 µM | | |
| Calcium pantothenate | 4.2 µM | | |
| Biotin | 0.4 µM | | |
| Folic acid | 2.3 µM | | |
| Niacinamide | 8.2 µM | | |
| L-alanine | 2.7 mM | | |
| L-glutamine | 2.7 mM | | |
| L-asparagine monohydrate | 2.7 mM | | |
| L-arginine | 0.7 mM | | |
| L-lysine monohydrochloride | 2.4 mM | | |
| L-isoleucine | 1.6 mM | | |
| L-methionine | 0.8 mM | | |
| L-phenylalanine | 1.7 mM | | |
| L-serine | 3.2 mM | | |
| L-threonine | 1.9 mM | | |
| L-tryptophane | 0.2 mM | | |
| L-valine | 2.8 mM | | |
| Glycine | 2.3 mM | | |
| L-histidine | 0.7 mM | | |
| L-leucine | 3.6 mM | | |
| DL-proline | 0.6 mM | | |
| L-cysteine | 1.7 mM | | |
| L-aspartic acid | 2.3 mM | | |
| Choline chloride | 36 µM | | |
| Adenine | 74 µM | | |
| Uracil | 89 µM | | |
| Sodium pyruvate | 91 µM | | |
|  |  | | |
|  | CDM | CDM+M | CDM+MS |
| Magnesium chloride hexahydrate | 1.0 mM | - | - |
| Calcium chloride | 0.3 mM | - | - |
| Zinc sulfide heptahydrate | 17 µM | - | - |
| Cobalt chloride hexahydrate | 11 µM | - | - |
| Copper(II) sulfate pentahydrate | 0.4 µM | - | - |
| Manganese (II) sulfate monohydrate | 166 µM | - | - |
| Manganese standard | - | 0.7 µM | 0.7 µM |
| Magnesium standard | - | 1.4 mM | 1.4 mM |
| Calcium standard | - | 1.7 mM | 1.7 mM |
| Cobalt standard | - | 0.7 µM | 0.7 µM |
| Copper standard | - | 6.5 µM | 6.5 µM |
| Zinc standard | - | 12 µM | 12 µM |
| Iron standard | - | 21 µM | 21 µM |
| glucose | 28 mM | 28 mM | 65 µM |
| fucose | - | - | 0.3 µM |
| GlcNAc | - | - | 0.03 µM |
| GalNAc | - | - | 5 µM |
| fructose | - | - | 0.3 µM |
| mannose | - | - | 0.2 µM |
| galactose | - | - | 0.05 µM |
| maltose | - | - | 11 µM |
| Neu5Ac | - | - | 12 µM |
| mucin from porcine stomach | - | - | 0.1 g/L |
