# Supplementary material for: Streptococcus pneumoniae adaptation to nutrient deprivation and immune modulation drives upper respiratory tract colonization
Source: ISME J. 2026 Aug 19;20(1):wrag213. doi: 10.1093/ismejo/wrag213 (PMC13561108; doi:10.1093/ismejo/wrag213)
Supplement: Supplementary file 1 [file supplementary_material_wrag213.zip › 26-08-25 Supplementary Table 5.docx]

**Sup. Table 5: nanoLC-MS/MS Settings**

| **Reversed phase liquid chromatography (RPLC)** | |  |
| --- | --- | --- |
| Instrument | Ultimate 3000 RSLC (Thermo Scientific) |  |
| Trap column | 75 μm inner diameter, packed with 3 μm C18 particles (Acclaim PepMap100, Thermo Scientific) |  |
| Analytical column | Accucore 150-C18, (Thermo Fisher Scientific)  25 cm x 75 μm, 2.6 μm C18 particles, 150 Å pore size |  |
| Buffer system | Binary buffer system consisting of 0.1% acetic acid in HPLC-grade water (solvent A) and 100% ACN in 0.1% acetic acid (solvent B) |  |
| Flow rate | 300 nL/min |  |
| Gradient | Linear gradient of solvent B from 2% up to 25% for intracellular proteome analysis |  |
|  | 0min-2%  2min-5%  10min-7%  70min-25%  75min-40%  77min-90%  83min-90%  85min-2%  95min-2%  gradient of solvent B for secretome analysis |  |
| Gradient duration | 120 min |  |
| Column oven temperature | 40°C |  |
| **MS/MS Intracellular proteome** | | |
| Instrument | Q Exactive HF mass spectrometer (Thermo Fisher Scientific) | |
| Electrospray | Nanospray Flex™ Ion Source | |
| Operation mode | Data-independent | |
| **Full scan properties** |  | |
| MS scan resolution | 60000 | |
| Normalized AGC target | 5e6 | |
| Maximum ion injection time for the MS scan | 200 ms | |
| Scan range | 333 to 1650 m/z | |
| RF Lens | 50 % | |
| Spectra data type | Profile | |
| **Dia properties (MS2)** |  | |
| Precursor mass range | 333 to 1650 m/z | |
| Resolution | 30,000 | |
| Norm. MS/MS AGC target | 3e6 | |
| Maximum ion injection time mode | Auto | |
| Spectra data type | Profile | |
| Microscans | 1 | |
| Isolation window | 56 windows, 13 m/z, 2 m/z overlap | |
| Define first mass | 200 | |
| Dissociation mode | Higher energy collisional dissociation (HCD) | |
| Normalized collision energy | 27.5 % | |
| **MS/MS Secretome** | | |
| Instrument | Orbitrap Exploris 480 mass spectrometer (Thermo Fisher Scientific) | |
| Electrospray | Nanospray Flex™ Ion Source | |
| Operation mode | Data-independent | |
| **Full scan properties** |  | |
| MS scan resolution | 120000 | |
| AGC target | 3e6 (300%) | |
| Maximum ion injection time for the MS scan | 60 ms | |
| Scan range | 350 to 1200 m/z | |
| Microscans | 1 | |
| Polarity | Positive | |
| RF lens | 50% | |
| Spectra data type | Profile | |
| **Dia properties (MS2)** |  | |
| Resolution | 30000 | |
| Maximum ion injection time for the MS/MS scans | Auto | |
| Normalized AGC target | 3e6 | |
| Spectra data type | Profile | |
| Microscans | 1 | |
| Isolation window | 66 | |
| Isolation window width | 13 m/z | |
| Window overlay | 2 m/z | |
| Fixed first mass | 200 | |
| HCD collision energy | 30% | |
